# Habitat characterization and species distribution model of the only large‐lake population of the endangered Silver Chub (*Macrhybopsis storeriana*, Kirtland 1844)

**DOI:** 10.1002/ece3.6830

**Published:** 2020-10-07

**Authors:** James E. McKenna, Patrick M. Kocovsky

**Affiliations:** ^1^ US Geological Survey Great Lakes Science Center Tunison Laboratory of Aquatic Science Cortland NY USA; ^2^ US Geological Survey Great Lakes Science Center Lake Erie Biological Station Sandusky OH USA

**Keywords:** aquatic habitat, Great Lakes, Lake Erie, native species, species distribution

## Abstract

The endangered Silver Chub (*Macrhybopsis storeriana,* Kirtland 1844) is native to North America and primarily riverine, with the only known large‐lake population in Lake Erie. Once a major component of the Lake Erie fish community, it declined and became nearly extirpated in the mid‐1900s. Recent collections in western Lake Erie suggest that Silver Chub may be able to recover, but their habitat and distribution are poorly known. A recent work showed an extensive area of western Lake Erie with the potential to support large numbers of Silver Chub, but was based on a geographically limited dataset. We developed a neural network‐based species distribution model for the Silver Chub in western Lake Erie, improved by new synoptic data and using habitat variables resistant to anthropogenic activities. The Potential model predictions were compared with a model that included anthropogenic‐sensitive variables. The Potential model used 10 habitat variables and performed well, explaining > 99% of data variation and had generally low error rates. Predictions indicated that a large area of the waters approximately 2–9 m deep contained Appropriate habitat and the highest abundances should be supported by habitat in a wide arc through the western end of the basin. The model indicated that Appropriate Silver Chub habitat was associated with relatively deep water, near coastal wetlands, where effective fetch is less than average. Disturbance model predictions were similar, but predicted poorer Silver Chub habitat in more areas than that predicted by the Potential model. Our Potential model reveals Appropriate habitat conditions for Silver Chub and its spatial distribution, indicating that extensive areas of western Lake Erie could support Silver Chub. Comparisons with Disturbance model predictions demonstrate that Potential model predictions may be used in conjunction with analyses of degrading conditions in the system to better conserve and manage for this endangered species.

## Graphical abstract

The only Great Lake population of Silver Chub (*Macrhybopsis storeriana*) is imperiled, and the species is listed as Endangered in parts of Lake Erie. The model developed in this study characterizes best potential Silver Chub habitat and its distribution in Western Lake Erie and compares them with disturbed conditions, supporting conservation of this unique population.

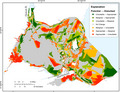

## 1 INTRODUCTION

The Silver Chub (*Macrhybopsis storeriana*) is a minnow native to central North America (Kinney, 1954; McCulloch et al., 2013). Most of its distribution is within riverine habitat of the Mississippi watershed but a large‐lake population also exists in Lake Erie (FishBase, 2014; McCulloch et al., 2013; ODNR, 2014; Page & Burr, 2011). Where it occurs, Silver Chub is consumed by piscivores (Hoover et al., 2007) and may historically have been a substantial component of the Lake Erie ecosystem. Kinney (1954) estimated that until the 1950s there were 8–80 Silver Chub/ha in western Lake Erie. This is similar to present densities of mimic shiners (*Notropis volucellus*) (USGS, 2018). However, the Lake Erie population declined severely in the late 1950s, possibly due to habitat degradation or reduced food availability (Kinney, 1954), and never recovered to its prior abundance (COSEWIC, 2012; Parker et al., 1987). After the 2012 Committee on the Status of Endangered Wildlife in Canada (COSEWIC) assessment, Silver Chub was designated as endangered in the Great Lakes‐St. Lawrence unit (COSEWIC, 2012). This species is also listed as endangered and is extirpated from New York waters of Lake Erie, listed as special concern [Critically Imperiled] in Michigan (Kočovský, 2019), and has not been collected in Pennsylvania waters of Lake Erie since the 1970s.

The first Silver Chub species distribution model (SDM) for western Lake Erie showed extensive areas of the basin with the potential to support large populations (McKenna & Castiglione, 2014). However, data were limited to collections before 2003 and did not include samples from Canadian waters. As a result, predictions for much of the habitat in western Lake Erie were based on extrapolation into areas that were not well represented within their dataset. McKenna and Castiglione (2014) also included some explanatory variables that are sensitive to human activities, which may reflect status appropriate to the time frame of the dataset, but is not as effective at providing the best benchmark of the habitat's potential to support Silver Chub.

Higher resolution data can improve the Silver Chub distribution model and enhance conservation of this species. We redeveloped the SDM for Silver Chub in western Lake Erie using a temporally and spatially more extensive dataset than was available to McKenna and Castiglione (2014) and restricted the explanatory variables to those least influenced by human activity. This allows for the best estimation of the potential for the lake's habitats to support Silver Chub, if human influence was minimized. This benchmark of potential (i.e., the highest Silver Chub abundance that can be expected) is critical to focus conservation on habitats that are the best candidates for protection or restoration, when predictions are compared with present‐day conditions (e.g., observations (McKenna & Johnson, 2011) or disturbance model predictions), and may guide scientific sampling efforts and population monitoring.

Our objectives in this study were to (a) train an artificial neural network model (NN) with the best available standardized trawl data and matching broadscale habitat conditions to predict Silver Chub abundances, (b) apply that model to predict the potential distribution of Silver Chub throughout western Lake Erie, (c) describe the habitat conditions associated with various qualities of Silver Chub habitat, and (d) contrast predictions with a model that includes disturbances and discuss some of the conservation implications of this improved model.

## 2 METHODS

Data on fish collected in western Lake Erie by trawling programs in Ohio and Ontario were provided by the Ontario Ministry of Natural Resources and Forestry (OMNRF), Lake Erie Management Unit, and the Ohio Department of Natural Resources (ODNR), Division of Wildlife, Lake Erie Fisheries Unit. These trawl data are fishery‐independent and included summer catches of Silver Chub, as well as the absences of the species and information about the location, gear, and fishing effort. Silver Chub catches were standardized to catch per unit effort (CPUE) in units of fish/1,000 m^2^ (assuming 100% catchability) and then categorized into a log‐scale of abundance classes, following the methods of McKenna and Castiglione (2014). The finest scale of the spatial framework used for this study is the 90‐m cell (McKenna & Castiglione, 2010, 2017). Habitat variable values and fish observation data were assigned to the spatial cells within which those data were collected. Coarser‐scale units are available in this spatial framework, including Aquatic Habitat Areas (AHA). All 90‐m cells within an AHA have similar basic fish habitat conditions, namely effective fetch (energy), distance from the nearest large river (i.e., Strahler ≥ 5 at the mouth) (material and water source), predicted presence of substantial submerged aquatic vegetation (SAV) (three‐dimensional structure), and water depth (habitat volume). Variability of fish abundance data was high due to the rarity of this species in the lake. Thus, individual CPUE values were averaged within the slightly coarser AHA spatial units. Classification of CPUE into the log‐scale classes (1, 2–10, >10) was based on these averages; we considered “Appropriate” conditions those that supported at least 2 fish/1,000 m^2^. A suite of lakescape data was provided at the 90‐m resolution for all spatial cells within the entire Western Lake Erie Aquatic Lake Unit (ALU) (a coarse‐scale spatial unit based on large‐scale circulation patterns) by the Great Lakes Regional Aquatic Gap Analysis Coastal Project (McKenna & Castiglione, 2010, 2014, 2017) (Table 1, Figure S1a–j and Figure S2a–i). While some data for variables such as water temperature and shoreline modification were available, we chose to use only variables that are not affected by human activity over ecological time scales (decades to centuries) to provide the best predictions of the potential that each habitat unit has to support Silver Chub.

**Table 1:** Summary statistics for important habitat variables in Western Lake Erie where fish samples were collected

Variable class	Variable [code]	Mean	Model relative weights	
			Potential	Disturbance
3‐D Structure	SAV covering ≥ 50% of bottom [GLSAV]]	0.094869	−1.029	0.125
Coastal Geomorphology	Bedrock that is resistant to erosion [ShoreNear_C04]	34.23136	−0.962	−0.281
	Baymouth‐barrier beaches [ShoreGeom_C09]	17.59342	−0.770	0.189
Distance to Coastal Habitat	Distance to the nearest Protected‐type Wetland (m) [GLWetProtDist]	9,989.768	5.337	0.235
	Distance to the nearest Delta‐type Wetland (m) [GLWetDeltaDist]	16,332.54	−9.652	0.712
	Distance to the nearest Open‐type Wetland (m) [GLWetOpenDist]	24,348.54	−0.706	−0.033
Habitat Volume	Depth (m) [GLBath90m]	−8.0485	0.194	0.314
Material Source	Number of major rivers (Strahler Order ≥ 5) entering the lake (km^−2^ × 10^6^)^a^ [GLRivDens]	169.8925	−2.585	0.048
	Distance to the nearest major river (m) [GLRivDist]	19,072.66	3.705	−0.004
System Energy	Effective fetch (m) [GLFetch]	21,895.14	5.467	0.040
Water Temperature	May surface water temperature (°C) [sst_May]	13.24		−0.012
	September surface water temperature (°C) [sst_Sep]	21.74		0.074
	CV of August surface water temperature [cv_Aug]	0.055		−0.162
	CV of September surface water temperature [cv_Sep]	0.081		0.102
Shoreline Modification^b^	Major shoreline modification (70−100%) [SM_1]	20.03%		−0.165
	Moderate shoreline modification (40−70%) [SM_2]	10.25%		0.103
	Minor shoreline modification (15−40%) [SM_3]	39.02%		−0.094
	Unmodified shoreline (<15%) [SM_4]	30.25%		0.095
	Nonstructural modifications [SM_5)	0.43%		−0.285

Note

Variable class is the broad type of habitat, and the Variable is the specific habitat that fish respond to. Mean is the mean value of the Variable. Model Relative Weights are the weight assigned by the neural network model to each variable. Coastal Geomorphology and 3‐D Structure have units of % of area covered. CV indicated coefficient of variation.

^a^ River density was calculated as the number of rivers of Strahler Order ≥ 5 at their mouths within a 100‐km radius circle from each spatial cell on a per km^2^ basis and multiplied by 106 to produce integer values for the raster representation of the data in the geographic information system.

^b^ Hillyer (1996)

### 2.1 Model of potential

We used a principal components analysis (PCA) on the habitat data to identify the variables most likely to influence the distribution and abundance of Silver Chub in Lake Erie. The influence of each variable was ranked based on the length of its vector in the plane of the first two axes. Decrease in the relative weight of ranked variables along the first two PCA axes suggested that 10 variables would explain most variation. Those top ten variables were then used to develop and train the NN model (NeuroShell 2.0 software, Wards Systems Group, Inc.).

We used a NN because of its ability to effectively represent complex pattern associations where nonlinear responses are likely to exist, and because NNs do not suffer from typical limitations of assumptions about the underlying response model and specific error structure (Hertz et al., 1991; McKenna, 2005; Olden & Jackson, 2001). We used a simple backpropagation model with one hidden layer of neurons and a logistic activation function. This model was trained to predict the observed abundance class of Silver Chub at each of the 2,066 locations where a trawl occurred. Twenty percent of the data were randomly selected and held out from the training as an internal test dataset to prevent overlearning. The influence of each habitat variable on predictions of Silver Chub abundance class was determined by tracing the changes in weightings through the final NN (McKenna, 2005; McKenna & Castiglione, 2014).

Model effectiveness and reliability were evaluated in several ways. First, the model was deemed acceptable if it explained at least 90% of the variability in the dataset (adjusted coefficient of determination [$R_{\text{adj}}^{2}$] and mean square error [*MSE*] are reported). Omission and commission error rates of the presence and absence predictions are also provided. In addition, Cohen's Kappa, a commonly used measure of model performance relative to chance predictions (*K* > 0.6 is substantial, Landis & Koch, 1977), was calculated for overall presence–absence predictions and for each abundance class. Also, visual inspection of the match between observed and predicted abundance classes was conducted on a map of the study area generated with a Geographic Information System (ArcGIS 10 [ESRI]). Signatures of the habitat conditions associated with each class of Silver Chub abundance were determined by mean *Z*‐scores of conditions within each class, relative to average conditions throughout the study area.

Additional independent test datasets were also available to evaluate model performance. The ODNR provided a dataset that included Silver Chub collections in spring (May and June) and fall (October), but did not include absences (hereafter OH data). The US Geological Survey (hereafter USGS data) provided a dataset that included both Silver Chub collections and absences (USGS, 2018), but the rareness and patchiness of this species made those data highly variable. In addition, differences in trawling methods were evident in the data. Thus, we used only samples where Silver Chub were present for model testing, because the differences in methods were substantial, compared to that which generated the data for model construction (ODNR and OMNRF summer samples). The low abundance class (1’s) collections were also excluded from these data, based on catchability differences and because the distribution of USGS low fish abundance collections was in areas where other data (OH data and model construction data) detected relatively high numbers. Thus, this stage of model testing focused on Moderate and high (Optimal) abundance collections and their match to model predictions, which is likely to highlight the habitats most appropriate for conservation. Both simple linear correlation and Kolmogorov–Smirnoff (K‐S) goodness‐of‐fit tests were used to evaluate the match between these observed test data and predicted abundance classes, as well as visual inspection of the collection points on the predictive map.

### 2.2 Disturbance Model and Contrasts

While our primary goal was to develop the model of best potential habitat conditions (hereafter Potential model) for Silver Chub throughout western Lake Erie, model predictions that incorporate some level of disturbance (Disturbance model) provide useful contrasts and help to illustrate the value of benchmark predictions provided by the Potential model. Many possible disturbances to Silver Chub habitat conditions exist in Lake Erie. However, only two classes of anthropogenically influenced variables were available to us for this study, surface water temperatures and artificial shoreline modification. Also, little information exists on the sensitivity of Silver Chub to various potentially degrading environmental factors (but see, Britt, 1955; Kinney, 1954; Krumholtz & Minckley, 1964). We are assuming that unnatural shoreline conditions and nonoptimal temperatures would adversely affect the Silver Chub distribution and would be reflected in habitat distributions. Mean surface water temperatures (°C) and their coefficients of variation (CV) were available in each 90‐m spatial cell of the study area for each month from May through October (Table S1). There was high autocorrelation among these variables, and only two temperature and two CV variables were required to represent those data and their variability (Table 1, Figures S2a–d). Each of the five shoreline protection variables expresses the proportion of spatial cells classified as having one of five types of shoreline modification. The Disturbance model was developed by modifying the Potential model with the addition of the two surface temperature variables, two surface temperature CV variables, and all five shoreline protection variables (Table 1). The same neural network learning procedure was used to train the Disturbance model as the Potential model. However, the number of hidden neurons was changed to a value appropriate to the increased number of input variables. Habitat predictions of the Potential and Disturbance models for each class of Silver Chub habitat quality were compared on a spatial cell‐by‐cell (90‐m) basis, and differences and similarities were tabulated and mapped. Habitat fragmentation can indicate the quality of natural environments for populations or communities, and many different measures of fragmentation exist (McGarigal & Marks, 1995; Turner, 1989). We selected five simple metrics of habitat distribution configuration and fragmentation appropriate for this study (Percentage of total area, Number of patches, Mean patch size, Mean distance to nearest similar patch, and Aggregation Index) to help compare and contrast model predicted Silver Chub habitat distributions.

## 3 RESULTS

**Figure 1:**
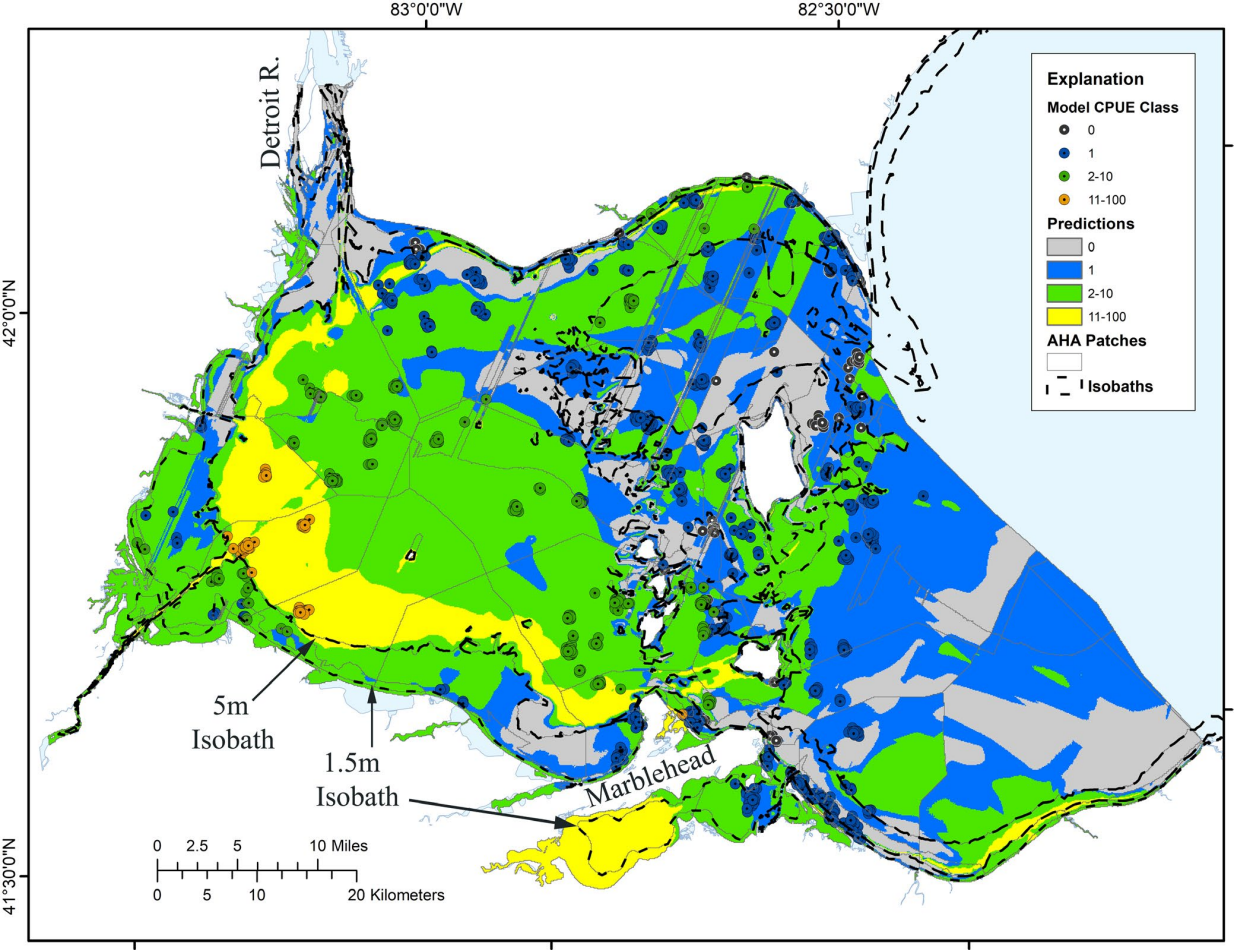
Ontario MNRF and Ohio DNR trawl collection sites (circles) and Potential model predictions (polygons) of silver chub abundance (number/1,000 m^2^) in Western Lake Erie. Abundance classes equate to Optimal (11–100/1,000 m^2^), Moderate (2–10/1,000 m^2^), Marginal abundances (1/1,000 m^2^), and unsuitable (0/1,000 m^2^). The light gray lines indicate AHA boundaries and dashed black lines indicate depth isobaths of 1.5, 5, and 10 m

Catches from 2,066 ODNR and OMNRF trawl samples from 1987 to 2014 collected 9,414 Silver Chub and were standardized and assigned to the appropriate log‐scale abundance classes. Silver Chub were collected in 676 samples and CPUE ranged from 0.32 to 503/1,000 m^2^ but was less than 4/1,000 m^2^ in 76% of the samples where Silver Chub was present. There was no trend in Silver Chub CPUE during the sampling time period. The catches were widely distributed throughout the western basin of Lake Erie and were usually in the same areas where they were absent from previous, subsequent, or adjacent trawls; the highest abundances occurred in the southwestern end of the lake in approximately 5.5–7.0 m of water (Figure 1). More than 99% of Silver Chub were collected in waters > 2.5 m deep.

The first two axes of the PCA explained 30.1% of variation in the data. Among the 29 anthropogenically resistant habitat variables available, the 10 most influential variables included representation of system energy, distance to major rivers and wetlands, coastal geomorphology, SAV, and habitat volume (Table 1, Appendix S1).

### 3.1 Model of best potential

#### 3.1.1 Model performance

The NN model of Potential used the 10 most influential habitat variables as inputs and 46 neurons in the hidden layer. The model performed well, explaining 99.5% of variation ($R_{\text{adj}}^{2}$ = 0.995, *MSE* = 0.64). Correct predictions of abundance classes were high (96%) (Table 2). On an overall presence–absence basis, the commission error rate was high (53%), but only a single omission error occurred. Cohen's Kappa shows that error rates for the model, both on the basis of presence–absence and for predictions of any given abundance class, were substantially lower than expected by chance and indicate a strong model. Visually, the match between observed abundance values and that predicted by the model was good (Figure 1).

**Table 2:** Correct predictions and omission and commission errors

a. Presence–absence model (prevalence = 93.1%, Potential model *K* = 0.62, Disturbance model *K* = 0.78)			
	Abundance Class	Observed	
		Absent	Present
Potential Model Predicted	Absent	67	**1**
	Present	*76*	1,922
Disturbance Model Predicted	Absent	95	**1**
	Present	*48*	1,922

b. Optimum abundance class (11–100) (prevalence = 5.8%, Potential model *K* = 1.00, Disturbance model *K* = 1.00)			
	Abundance Class	Observed	
		Not > 10	>10
Potential Model Predicted	Not > 10	1,947	**0**
	>10	*0*	119
Disturbance Model Predicted	Not > 10	1,947	**0**
	>10	*0*	119

c. Moderate abundance class (2 10) (prevalence = 25.7%, Potential model *K* = 0.88, Disturbance model *K* = 0.98)			
	Abundance Class	Observed	
		Not 2–10	2–10

Potential Model Predicted	Not 2–10	1,435	**1**
	2–10	*100*	530
Disturbance Model Predicted	Not 2–10	1,524	**2**
	2–10	*11*	529

d. Marginal abundance class (1) (prevalence = 61.6%, Potential model *K* = 0.83, Disturbance model *K* = 0.95)			
	Abundance Class	Observed	
		Not 1	1
Potential Model Predicted	Not 1	724	**93**
	1	*69*	1,180
Disturbance Model Predicted	Not 1	750	**5**
	1	*43*	1,268

Omissions are in bold, and commissions are in italics. Cohan's Kappa (*K*) and prevalence are also shown for each model and abundance class.

#### 3.1.2 Model predictions

The Potential model predicted a mosaic of habitats throughout the western basin of Lake Erie capable of supporting various abundances of Silver Chub (Figure 1). Appropriate habitat (i.e., the two largest abundance classes, Optimal (>10/1,000 m^2^) and Moderate (2–10/1,000 m^2^)) was predicted to occupy > 50% of the Western Lake Erie Aquatic Lake Unit area, of which only 10% was expected to support Optimal densities. Optimal habitat conditions were predicted to generally occur in a band from the Marblehead Peninsula to the mouth of the Detroit River, in waters about 6.5 m deep. Most of the study area was encompassed by habitat predicted to have the potential to support Moderate abundance of Silver Chub, which occupied western, central, and northern portions of the lake unit, plus Sandusky Bay. Nearly 1/3 of the study area was predicted to support Marginal abundances (1/1,000 m^2^) of Silver Chub, and only 17% of the area was predicted to be Unsuitable. Most of the Marginal and Unsuitable habitats were located in the central and eastern portions of the lake unit. A complex mosaic of habitat patches existed around the archipelago that roughly separate the western and central basins of Lake Erie and along the southeastern coast of the western basin. The Detroit River Delta in the northwestern portion of the lake is the primary conduit of water entering Lake Erie and was predicted to be largely Unsuitable.

The Silver Chub abundance classes increase in size as abundance increases, and the model provides only broad relative abundance estimates. Model predictions could be used to provide a range for the potential Silver Chub population size within the western Lake Erie Aquatic Lake Unit (Table 3). While such an estimate would be similar to Kinney's original estimate (Kinney, 1954), capture variability and other uncertainties make such an estimate highly speculative.

**Table 3:** The number of 90‐m spatial cells (Potential model allocation), areal extent, and percentage of the study area predicted by the Potential or Disturbance models to support each class of Silver Chub habitat quality

Habitat quality	90‐m cells	Potential model Area (km^2^) (% ALU)	Disturbance model Area (km^2^) (% ALU)	Minimum population estimate	Maximum population estimate
Optimal (>10/1,000 m^2^)	58,054	470 (10%)	726 (16%)	4,937,493	47,023,740
Moderate (2–10/1,000 m^2^)	240,740	1,950 (43%)	1,649 (36%)	2,924,991	19,499,940
Marginal (1/1,000 m^2^)	167,210	1,355 (30%)	1,170 (26%)	1,354,401	1,354,401
Unsuitable (0/1,000 m^2^)	95,801	776 (17%)	1,006 (22%)	0	0
Total	561,805	4,551	4,551	9,216,885 ± 30%	67,878,081 ± 4%

Note

Total Potential model predicted silver chub population range for the western Lake Erie Aquatic Lake Unit (ALU). Error estimates around population totals are based on the root mean squared error estimate from fit of the neural network model.

#### 3.1.3 Habitat characteristics

**Figure 2:**
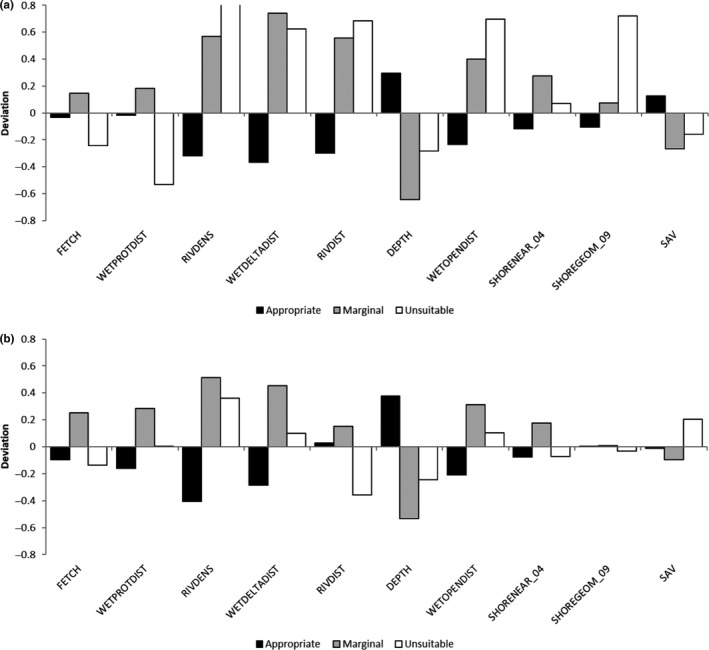
Habitat signatures by habitat quality type based on (a) only habitat conditions at locations where fish samples were collected and classified by observed Silver Chub abundance class, and (b) habitat conditions at all spatial locations in the study area and classified by Potential model Silver Chub abundance class predictions. Bar height represents *z*‐score deviations from the model development dataset average for conditions associated with Appropriate (black), Marginal (gray), and Unsuitable (open bars) silver chub habitats. See Table 1 for habitat variable definitions

Appropriate Silver Chub habitat may be described by environmental conditions observed at the subset of sample locations or throughout the study area, based on NN model interpolation and extrapolation to all habitats within the ALU; both representations are provided here (Figure 2). In addition, tracing habitat variable weightings through the NN provides insight into the workings of the model and a representation of habitat conditions associated with each Silver Chub abundance class (Figure S3). The Potential NN model placed the heaviest weight on distance to delta‐type (−) and protected‐type (+) wetlands, distance to large rivers (+), and effective fetch (+) (Table 1). Habitat signatures based on model extrapolation better represent the full range of habitat conditions than that based on only conditions at locations of fish samples (Figure 2a), although both were quite similar. The signature of Appropriate habitat conditions (i.e., those with the potential to support ≥ 2 Silver Chub/1,000 m^2^) based on the synoptic predictions of the Potential model emphasized relatively deep water, near coastal wetlands, where effective fetch is less than average and few major rivers enter the lake (Figure 2b). Marginal habitats were in shallow waters where effective fetch is large, relatively far from coastal wetlands and major rivers with little SAV, but adjacent to shores where several large rivers enter the lake and the shoreline sediment is mostly erosion‐resistant bedrock. Unsuitable conditions were generally intermediate between Appropriate and Marginal habitat types, but relatively near a major river and where SAV is abundant.

#### 3.1.4 Prediction evaluations

**Figure 3:**
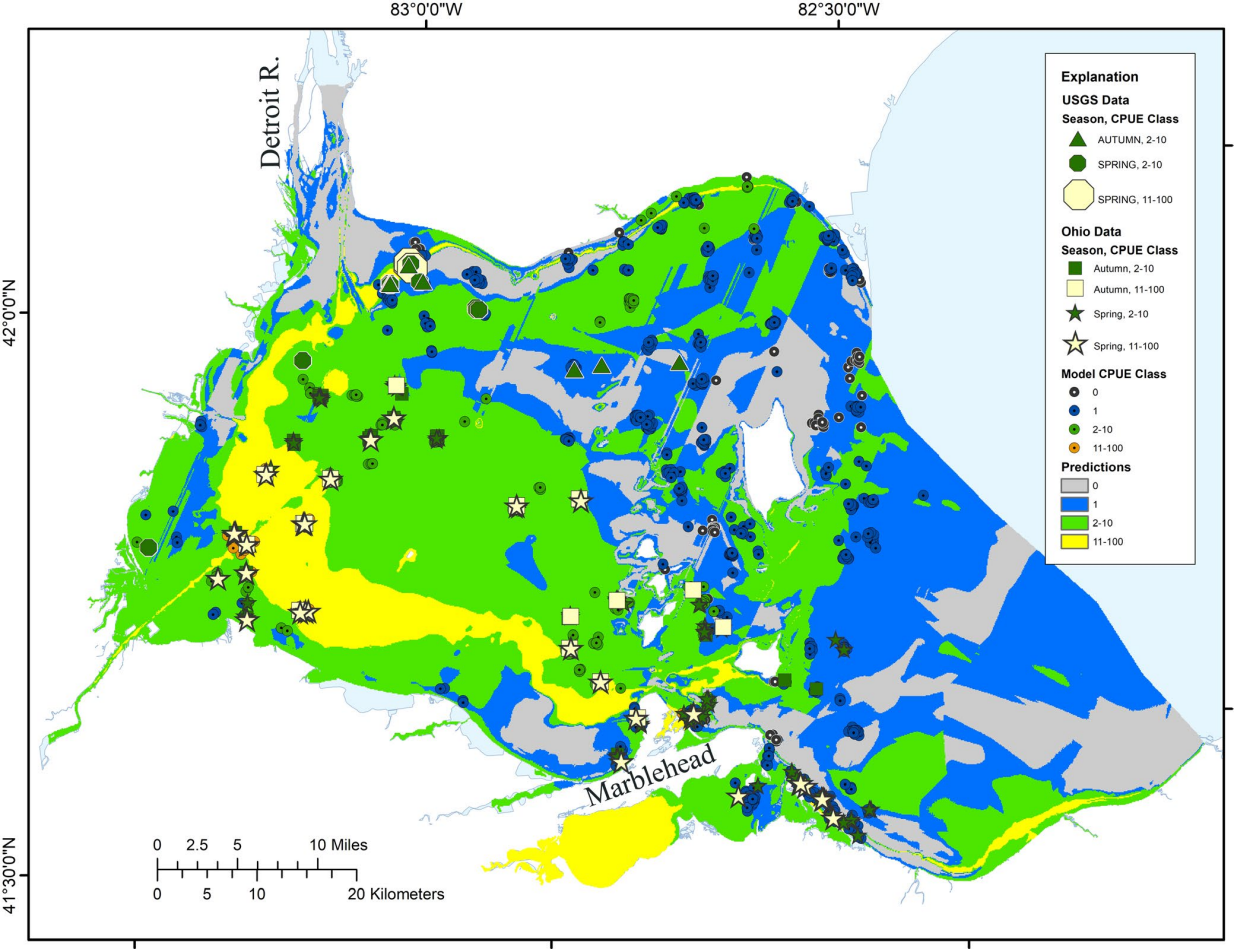
Ontario MNRF and Ohio DNR trawl collection samples used in model development (centered circles), spring and fall samples from the USGS (circles and triangles) and Ohio DNR (squares and stars), and Potential model predictions (polygons) of silver chub abundance (number/1,000 m^2^) in Western Lake Erie. Abundance classes equate to Optimal (11–100/1,000 m^2^), Moderate (2–10/1,000 m^2^), Marginal abundances (1/1,000 m^2^), and unsuitable (0/1,000 m^2^). Note that the symbols for the highest abundance classes for each group are stacked on top of numerous lower abundance symbols in some locations

Simple correlations between independent test data (OH data and USGS data) and predicted abundance classes were low for both datasets (OH: *r* = .10, USGS: *r* = .24). There were mixed results with goodness‐of‐fit tests (Table 4). The K‐S test with the Moderate and Optimal abundance USGS collections showed no significant difference with model predictions (i.e., a good match, *p* < .05), but for OH collections, indicated a poorer match to model predictions (*p* > .05). Visual observations of the spatial distributions of collections and predicted abundance classes revealed that there was generally a good spatial match between Moderate and Optimal abundance collections and model predictions, with several cases of a Silver Chub collection point being spatially close to a model prediction of matching abundance (Figure 3). Silver Chub were concentrated in the western portion of the western basin, with particularly high concentrations outside and east of the Detroit River Delta. Visual inspection of the distribution of Silver Chub collections in spring through fall also revealed little or no seasonal shift in the abundance of this species.

**Table 4:** Frequencies of Moderate and high abundance OH and USGS collections of silver chub within low and high predicted abundances classes and K‐S test results; *n* and critical *D* values are provided below each dataset name

Dataset	Predicted Abundance Class	Observed abundance class frequency	Expected abundance class frequency	Relative Expected abundance class frequency	D
OH Data	0–1	0	75	0.19	0.19
(*n* = 391, *D*_0.05,304_ = 0.069)	>1	4,835	316	0.81	
USGS Data	0–1	0	5	0.19	0.22
(*n* = 23, *D*_0.05,304_ = 0.28)	>1	250	18	0.81	

### 3.2 Disturbance model

#### 3.2.1 Model performance

**Figure 4:**
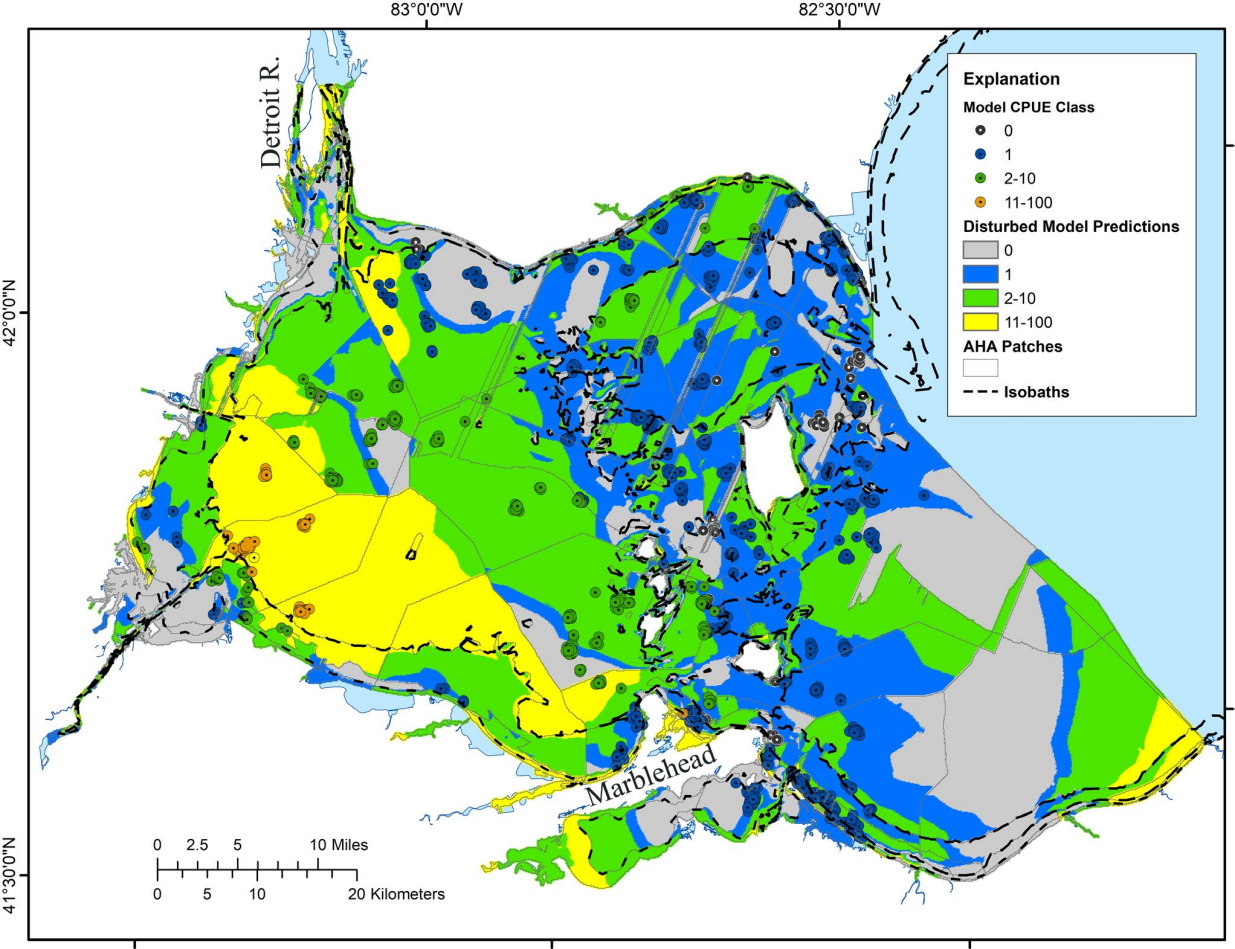
Ontario MNRF and Ohio DNR trawl collection sites (circles) and Disturbance model predictions (polygons) of silver chub abundance (number/1,000 m^2^) with effects of some anthropogenically influenced variables in western Lake Erie. Abundance classes equate to Optimal (11–100/1,000 m^2^), Moderate (2–10/1,000 m^2^), Marginal abundances (1/1,000 m^2^), and unsuitable (0/1,000 m^2^). The light gray lines indicate AHA boundaries, and dashed black lines indicate depth isobaths of 1.5 m, 5 m, and 10 m. Note that only shoreline modifications and water temperature variables represent disturbance effects.

The Disturbance model used the 10 habitat variables from the Potential model, plus the four temperature metrics and five shoreline modification variables as inputs and 55 hidden layer neurons. Model weightings were generally similar to those of the Potential model, except for distances to delta‐type wetlands and nearest larger river (Figure S3). The model performed well, explaining 99.9% of variation ($R_{\text{adj}}^{2}$ = 0.999, *MSE* = 0.05). Correct predictions of abundance classes were high (98%) (Table 2), and on an overall presence–absence basis, the commission error rate was relatively low (34%); only a single omission error occurred. Cohen's Kappa showed that error rates for the model, both on the basis of presence–absence and for predictions of any given abundance class, were substantially lower than expected by chance, indicating a strong model. Visually, the match between observed abundance values and model predicted values was good (Figure 4).

#### 3.2.2 Model comparisons

There were strong similarities between the Potential and Disturbance model predictions. Half (50.1%) of the study area was predicted to have the same Silver Chub habitat quality by both models (Table 5). Of the other half of the study area, most (>54%) was predicted to support poorer conditions under the added influence of the disturbance variables. However, in both models, the large area of Optimal habitat was concentrated in the western part of the ALU and most of the other Appropriate habitat (Moderate abundance class) surrounded that area, with the most extensive patch offshore to the east. A complex mosaic of mostly Marginal and Unsuitable habitat patches existed around the central island archipelago and eastward. The Disturbance model predicted more Optimal habitat (6%), but less Moderate habitat (7%) than the Potential model, resulting in prediction of nearly the same area of Appropriate (Optimal and Moderate abundance classes) habitat by both models. The Disturbance model also predicted less Marginal habitat and more Unsuitable habitat than the Potential model. The Disturbance model predicted somewhat higher quality habitat in parts of the Detroit River Delta than the Potential model, but predicted several embayments, notably Maumee Bay and Sandusky Bay, to be Unsuitable or of poorer quality. Among the fragmentation metrics, overall regional diversity of habitats across the ALU was slightly greater in the Disturbed (1.34) than in the Potential (1.26) model (Table 6). However, there were fewer patches of Appropriate and Marginal habitats and more patches of Unsuitable habitat in the Disturbance model and those Unsuitable habitats were slightly more aggregated. The mean patch size was larger for Optimal and Unsuitable habitats and smaller for other habitats in the Disturbed model than in the Potential model, while mean distance between patches of Appropriate habitats was greater in the Disturbed model. Mean distances between patches of other habitat types were approximately equal.

**Table 5:** Extent of areas predicted to have different Silver Chub habitat quality in the Disturbance model compared with the Potential model. Half (50.1%, 2,280 km^2^) of the study area was predicted to have the same habitat quality class in both models

Predicted Habitat Quality Class		Area affected (km^2^)	% of area
Potential Model	Disturbance Model		
Appropriate	Unsuitable	404.3034	8.9
Appropriate	Marginal	449.874	9.9
Marginal	Unsuitable	381.0483	8.4
Unsuitable	Marginal	225.8766	5.0
Marginal	Appropriate	479.1231	10.5
Unsuitable	Appropriate	329.8158	7.2

**Table 6:** Habitat fragmentation metrics for each habitat quality type comparing the predictions of the Potential (10 variable) and Disturbed (19 variables) Silver Chub models

Patch quality	% of total area		# of patches		Mean patch size (m^2^)		Mean distance (m)		Aggregation Index (%)	
	Potential	Disturbed	Potential	Disturbed	Potential	Disturbed	Potential	Disturbed	Potential	Disturbed
Optimal	10.2	16.0	142	162	326	448	559	663	96.1	96.9
Moderate	43.0	36.2	343	306	571	539	308	323	96.1	95.9
Marginal	29.8	25.7	618	594	219	197	281	288	94.4	94.1
Unsuitable	17.1	22.1	190	241	408	417	469	468	94.9	95.3

Note

Mean distance is the mean Euclidean distance (m) to the nearest neighboring patch of the same type, based on shortest edge‐to‐edge distance. Aggregation Index is basically the ratio of the number of like adjacencies to the maximum possible like adjacencies (see McGarigal & Marks, 1995).

Areas predicted by the Disturbance model to have lesser quality Silver Chub habitat than that predicted by the Potential model may have been associated with higher temperatures in May and higher water temperature variability (CVs) in August and September, but slightly lower water temperatures in September (Table 5, Figure S4a–d). Those areas also may have been associated with more extensive areas of major (70%–100%) or minor (15%–40%) shoreline modification (Figure S4e,g). However, values of each “disturbance” variable showed wide variability within classes of difference between the Potential model and Disturbance model. Means and medians of areas differing between model predictions were rarely more extreme than that associated with areas predicted to be the same quality by both models (Figure S4).

## 4 DISCUSSION

Only 10 anthropogenically resistant lakescape variables were needed to develop a NN model that effectively predicted the distribution of habitats with the potential to support various abundances of Silver Chub. Moderate and Optimal abundance collections consistently occurred within the areas predicted to support those high abundances, as evident from comparisons with both data used to construct the NN model and independent test data. A wide area within the Western Lake Erie ALU may be appropriate for Silver Chub, with a band of highest quality habitat arcing through the western end. The Potential model reveals that Appropriate habitat conditions are associated with relatively deep waters in proximity to coastal wetlands, where system energy is relatively low. Silver Chub occupy riverine habitat throughout most of their range, but are known to use low energy habitats within those lotic systems (e.g., Pflieger, 1975; Ross, 2001); whether the Lake Erie Silver Chub population is genetically distinct from riverine populations or would have different behaviors is unknown (but see Ahmad, 2017). Model predictions indicate that the majority of Lake Erie habitats have the potential to provide Appropriate or at least Marginal conditions for Silver Chub.

### 4.1 Performance of potential model

The more extensive dataset used here, particularly with representation from Canadian waters, allowed for improvement over the model developed by McKenna and Castiglione (2014). The metrics of model performance were all better than they report for their model, and the new model shows finer spatial detail, particularly in the northern part of the lake unit, which was strictly extrapolation in the previous model. The result is a more accurate description of the potential spatial distribution and extent of high‐ and low‐quality habitats with respect to the needs of Silver Chub within the lake unit and a better tool to support Silver Chub conservation efforts. However, differences in trawling vessels, when and where trawls occurred, and different catchabilities may affect prediction accuracy.

Our Potential model, like all models, has its limitations and, in addition to data changes, differs in a number of ways from the previous Silver Chub SDM for western Lake Erie (McKenna & Castiglione, 2014). Our model is based only on trawl data. Coastal electrofishing rarely collected Silver Chub (and most of those were around the islands), and no new electrofishing data were available for this project. Thus, we chose not to include data from that quite distinct collection method. Virtually all trawl collections occurred in waters > 0.5 m deep and the vast majority in much deeper water. Thus, model predictions in the shallowest waters are extrapolations. These shallowest areas are generally small, but upper Sandusky Bay stands out as an area predicted to have the potential to provide the highest quality habitat, in contrast to McKenna and Castiglione (2014). Anthropogenic influences on Sandusky Bay make it unlikely that that habitat is in its best possible condition and collections are needed to validate or refute the model predictions. Our Potential model predicted Unsuitable conditions in the Detroit River Delta. However, relatively high Silver Chub catches have occurred just outside of the delta in spring within the band of predicted Optimal habitat (USGS, 2018). Silver Chub are thought to spawn in open water, but may move nearshore or use tributary habitat (Goodyear et al., 1982; Kinney, 1954) and the delta and other tributaries may be important, if Appropriate habitat is available. Examination of locations of Moderate and Optimal abundance collections of Silver Chub in different seasons showed little spatial difference in the Silver Chub distribution. Thus, the model constructed with summer samples should provide reasonably good predictions of Silver Chub abundance from at least May through October. However, more research is needed on their seasonal movements.

#### 4.1.1 Model interpretation

The Potential model was designed to predict the best potential for any given habitat unit to support Silver Chub, and anthropogenic influences were explicitly excluded as inputs to provide that benchmark. The model relies on the large dataset to separate the signal of Silver Chub abundances matched to “natural” habitat conditions from the noise imposed by anthropogenic influences and other environmental and ecological factors (e.g., seasonal and other natural variation in the data and biological factors). To assist the model with this separation of signal from noise, we used averages of abundances and habitat conditions within AHAs, which helps to more clearly separate the array of habitat conditions associated with a particular class of Silver Chub abundance, but also reduces the resolution of predictions. Despite this generalization, we believe the model predictions are appropriate for most conservation efforts. In fact, they may be more appropriate than numerically specific predictions; one does not usually need to know if there are 7 versus 12 fish in a given area but rather are the fish rare, uncommon, common, or abundant—these qualitative classes match the model's quantitative abundance classes. The model predicts a temporal snapshot of Silver Chub habitat potential and does not explicitly capture seasonal or long‐term temporal dynamics, although no seasonal or long‐term trends were evident in the data (Figure 3).

### 4.2 Model comparisons

Habitat conditions may be driving differences in the extent of and locations that could support high or low Silver Chub abundances, and>¼ of the study area (27%) was predicted to support poorer conditions when effects of disturbance variables were included. However, direct relationships between independent variables and model predictions are difficult to make. We provide a cursory analysis of the associations of individual disturbance variables with differences in predictions between the two models (Figure S4). These suggest only possible weak associations between areas predicted to be of poorer quality and higher spring temperatures, summer temperature variabilities, and relatively extensive shoreline modifications; univariate relationships provide little insight into factors explaining differences. Neural networks use complex weighted combinations of all these variables to learn what best distinguishes each class of habitat quality. Weak associations of poorer quality conditions with the added influence of disturbance variables contribute to discriminatory power among habitat types when combined in the NN. However, NNs remain correlative and experimentation is required to determine cause and effects responses of Silver Chub.

Spatial distributions of any habitat type may be consolidated or fragmented. Fragmentation is often associated with degraded ecological conditions in terrestrial systems (Saunders et al., 1991; Turner, 1989; Wilcox & Murphy, 1985), but has rarely been examined quantitatively in aquatic systems (Jacobus & Webb, 2005). Subtle differences in the synergies within the two models can also affect predicted fine‐scale spatial distributions. Predictions of the Disturbance and Potential models were generally similar but with slightly different spatial distributions of the Optimal and Moderate habitat classes, resulting in nearly the same amount of Appropriate Silver Chub habitat in both models. Differences in fragmentation of model predictions, based on the selected metrics, were equivocal. The Disturbance model predicted fewer good‐quality and more poor‐quality habitats (which were more aggregated) and greater distance between good‐quality habitats, but greater overall patch diversity and larger good‐quality patches. As mentioned above, most habitat was predicted to be of equal or lesser quality by the Disturbance model than by the Potential model, but a minority of habitat was predicted to support higher quality Silver Chub habitat when the "disturbance" variables were included. The reasons for these predictions of improvements are not clear, but several factors should be considered with our example. It is possible that addition of the disturbance variables to the anthropogenically resistant variables of the Potential model revealed some higher quality conditions than what was detected by the learning process of the Potential model. However, some of these differences may be explained by minor geographic discrepancies between predictions, where one model predicted a certain quality condition to occur in a particular habitat patch, but the other model predicted that same habitat quality to occur in an adjacent location. This can be seen among the fragmented habitats around the islands. Another important factor is that some of the "disturbance" variables, like extent of unmodified shoreline, might be expected to indicate high‐quality (with large values) rather than low‐quality conditions. Finally, ecology and physiology of this rare species are poorly known, as are responses of Silver Chub to the disturbance variables used here (Kinney, 1954; Krumholtz & Minkley, 1964). For example, Silver Chub could prefer warmer water temperatures or higher variability in temperatures at certain times of the year, which would only be reflected in Disturbance model predictions. There is a large suite of stressors that degrade aquatic habitat in western Lake Erie (Hartig et al., 2009), and fragmentation associated with disturbances may become more evident with a more thorough representation than the two types included in our Disturbance model.

There were some general similarities between predictions of our models and those of the McKenna and Castiglione (2014) model, which generally predicted relatively uniform areas of Appropriate habitat offshore (derived from trawl data) and Marginal or Unsuitable habitats in nearshore areas (derived from electrofishing data). All three predicted the highest quality habitat to occur in offshore waters of the westernmost region of the ALU. Our Disturbance model predictions for large embayments were somewhat more like those of the McKenna and Castiglione (2014) model than predictions of the Potential model. However, extrapolation and differences in resolution, methods, and data between the studies make fine‐scale comparison difficult.

While the Potential model allows for an estimate of the potential western Lake Erie Silver Chub population (Table 3), the true present population is likely to be smaller due to degradation of habitats throughout the lake unit. This is a likely cause of the relatively high commission error rate of the Potential model. Widespread degradation might also limit the range of habitat qualities represented in the dataset; historically, there might have been habitat conditions that supported higher abundances. The NN models can only learn what is best within the dataset, and thus, our model predictions of best conditions are inherently conservative. The real value of the Potential model is as a tool to assist managers with identification of habitats that may be candidates for protection or restoration, by comparison of the benchmark prediction of a habitat's potential with observed present‐day conditions (represented to a small degree here by our Disturbance model predictions).

### 4.3 Conservation applications

By most definitions, our models are Species Distributions Models but, they have elements of Ecological Niche Models (ENM) (although representing a limited number of niche dimensions) by relating Silver Chub spatial distribution to its ecology (Peterson & Soberón, 2012), which can be valuable in conservation planning. However, conservation decisions without the benefit of ENM (or SDM) insights are common for various reasons (Tulloch et al., 2016) and can lead to protection or restoration efforts in inappropriate habitats. The benchmark of potential predicted by our model is extremely important, providing a means to determine how degraded a habitat is and whether or not the area is worthy of rehabilitation, but must be compared with present conditions to be most useful for conservation. Conservation decisions using only “reference” model predictions can have poor results, if areas selected for protection are, in fact, degraded (Hermosoa et al., 2011). Similarly, simply knowing present conditions (e.g., Disturbance model predictions), whether they are characterized as degraded or least‐disturbed, does not provide a measure of how greatly different they might be from the best conditions that could be achieved with habitat rehabilitation. Considering “present” conditions only is likely to exclude areas that may substantially improve success of conservation objectives with reasonable investments in rehabilitation.

**Figure 5:**
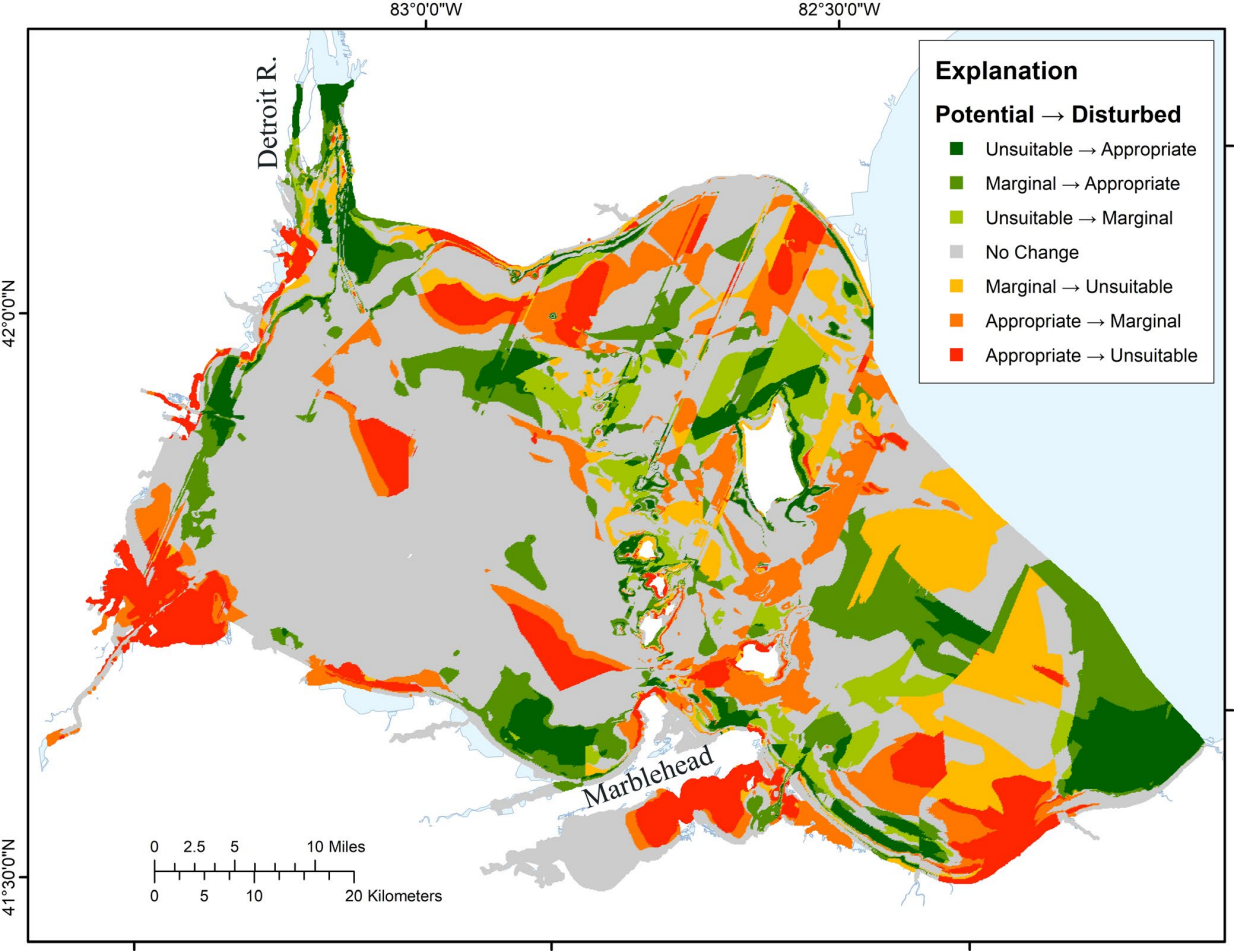
Differences in Potential and Disturbance model predictions of Silver Chub habitat in western Lake Erie. Differences are shown as changes from the habitat quality class predicted by the Potential model to that predicted by the Disturbance model. Red patches indicated the most negative difference between the Potential model and the Disturbance model, and darkest green indicates the most positive differences. Note that only shoreline modifications and water temperature variables represent disturbance effects

Comparisons of the benchmark reference and present‐day conditions are key to effective evaluation of protection or restoration opportunities. For example, opportunities for protection might occur where both of our models agree on Optimal conditions. Silver Chub feed on aquatic invertebrates, specializing on *Hexagenia* mayflies (Kocovsky, 2019), which burrow in soft benthic sediments. If a portion of the predicted highest potential habitat outside of Maumee Bay was found to be in excellent condition, that area might be a candidate for protection from dredging or other activities that disturb the benthos. Similarly, if Appropriate habitat is predicted by the Potential model to occur in an embayment that has been degraded that area may be a candidate for restoration. The Disturbance model predicts that most of Maumee Bay and lower Sandusky Bay are Unsuitable habitat for Silver Chub, but the Potential model predicts that those areas should be Appropriate, suggesting that rehabilitation of those habitats might benefit Silver Chub (Figure 5). In contrast, if an area predicted by the Potential model to be Unsuitable for Silver Chub is degraded, it makes no sense to expend resources to rehabilitate that area, with regard to conservation of Silver Chub.

## 5 CONCLUSIONS

Our improved Silver Chub model predictions help to address the need in the Canadian species recovery strategy for knowledge and a map of the current distribution and extent of suitable Silver Chub habitat to support conservation and future targeted sampling efforts for this species (McCulloch et al., 2013). Our benchmark predictions within the multiscale spatial framework give a better sense of the potential for this species under the best of circumstances than the previous model and provide for accounting of habitat quality and spatial distribution from fine‐ to coarse‐scale (McKenna & Castiglione, 2010). While comparisons of Potential and Disturbance model predictions help identify the degree of degradation in any habitat unit, additional models that include the broader array of disturbance variables and more accurately represent present‐day conditions can be developed and compared with benchmark (and Disturbance model) predictions developed in this study. Our empirically based modeling approach statistically describes the best conditions that might be achievable to support Silver Chub. This approach may also be applied to any other species, and multiple species comparisons could enhance conservation planning. Clearly, more research is needed on Silver Chub tolerances and the effects of other disturbance factors, and achieving remediation of degrading factors in Lake Erie is a high challenge. Neither model considers all habitat‐degrading factors, biological impediments, or socio‐political concerns, but they may be used with other tools and data to conduct triage and establish priorities for conservation of Silver Chub. Application of our predictions requires field validation and careful consideration of the factors not included in the models that may significantly affect Silver Chub abundance.

## CONFLICTS OF INTEREST

The authors declare no conflicts of interest.

## AUTHOR CONTRIBUTION

**James McKenna:** Conceptualization (equal); Data curation (supporting); Formal analysis (lead); Methodology (lead); Software (lead); Writing‐original draft (lead); Writing‐review & editing (equal). **Patrick Kocovsky:** Conceptualization (equal); Data curation (lead); Formal analysis (supporting); Writing‐original draft (supporting); Writing‐review & editing (equal).

## Supporting information

Supplementary Material

Appendix S1

## Data Availability

Most of the Silver Chub observation data from the US Geological Survey, Lake Erie Biological Station, are available from the USGS Great Lakes Science Center RVCAT database: https://doi.org/10.5066/F75M63X0. Ohio Department of Natural Resources and Ontario Ministry of Natural Resources and Forestry data reside in their interagency database available upon request to ODNR (Mark.Dufour@dnr.state.oh.us) or OMNRF (Zachary.Slagle@dnr.state.oh.us).
