# Supplementary material for: Habitat characterization and species distribution model of the only large‐lake population of the endangered Silver Chub (Macrhybopsis storeriana, Kirtland 1844)
Source: Ecol Evol. 2020 Oct 7;10(21):12076–90. doi: 10.1002/ece3.6830 (PMC7663989; doi:10.1002/ece3.6830)

Supplementary Tables and Figures.

Table S1. Pearson Correlation values among western Lake Erie surface waters by month from May through October and their coefficients of variation. Surface temperature is indicated by sst_ and coefficient of variation is indicated by cv_. Correlations of r > 0.5 are shown in bold. Temperature data source: McKenna and Castiglione (2017)

| Variable | cv_July | cv_June | cv_May | cv_October | cv_September | sst_August | sst_July | sst_June | sst_May | sst_October | sst_September |
| --- | --- | --- | --- | --- | --- | --- | --- | --- | --- | --- | --- |
| cv_August | 0.367 | 0.196 | 0.211 | 0.096 | 0.403 | -0.482 | -0.179 | -0.204 | -0.180 | -0.500 | **-0.934** |
| cv_July |  | **0.932** | **0.945** | **-0.855** | **-0.606** | **-0.980** | **-0.970** | **-0.938** | **-0.923** | **0.549** | -0.354 |
| cv_June |  |  | **0.997** | **-0.945** | **-0.804** | **-0.878** | **-0.978** | **-0.999** | **-0.999** | **0.726** | -0.102 |
| cv_May |  |  |  | **-0.948** | **-0.790** | **-0.897** | **-0.984** | **-0.994** | **-0.996** | **0.720** | -0.128 |
| cv_October |  |  |  |  | **0.923** | **0.767** | **0.945** | **0.940** | **0.951** | **-0.894** | -0.157 |
| cv_September |  |  |  |  |  | 0.476 | **0.764** | **0.792** | **0.817** | **-0.983** | **-0.506** |
| sst_August |  |  |  |  |  |  | **0.929** | **0.885** | **0.864** | -0.412 | **0.501** |
| sst_July |  |  |  |  |  |  |  | **0.981** | **0.972** | **-0.707** | 0.155 |
| sst_June |  |  |  |  |  |  |  |  | **0.996** | **-0.711** | 0.118 |
| sst_May |  |  |  |  |  |  |  |  |  | **-0.742** | 0.077 |
| sst_October |  |  |  |  |  |  |  |  |  |  | **0.575** |
|  |  |  |  |  |  |  |  |  |  |  |  |

Fig. S1. a – j. Western Lake Erie maps of values of variables used in the Silver Chub Potential model.


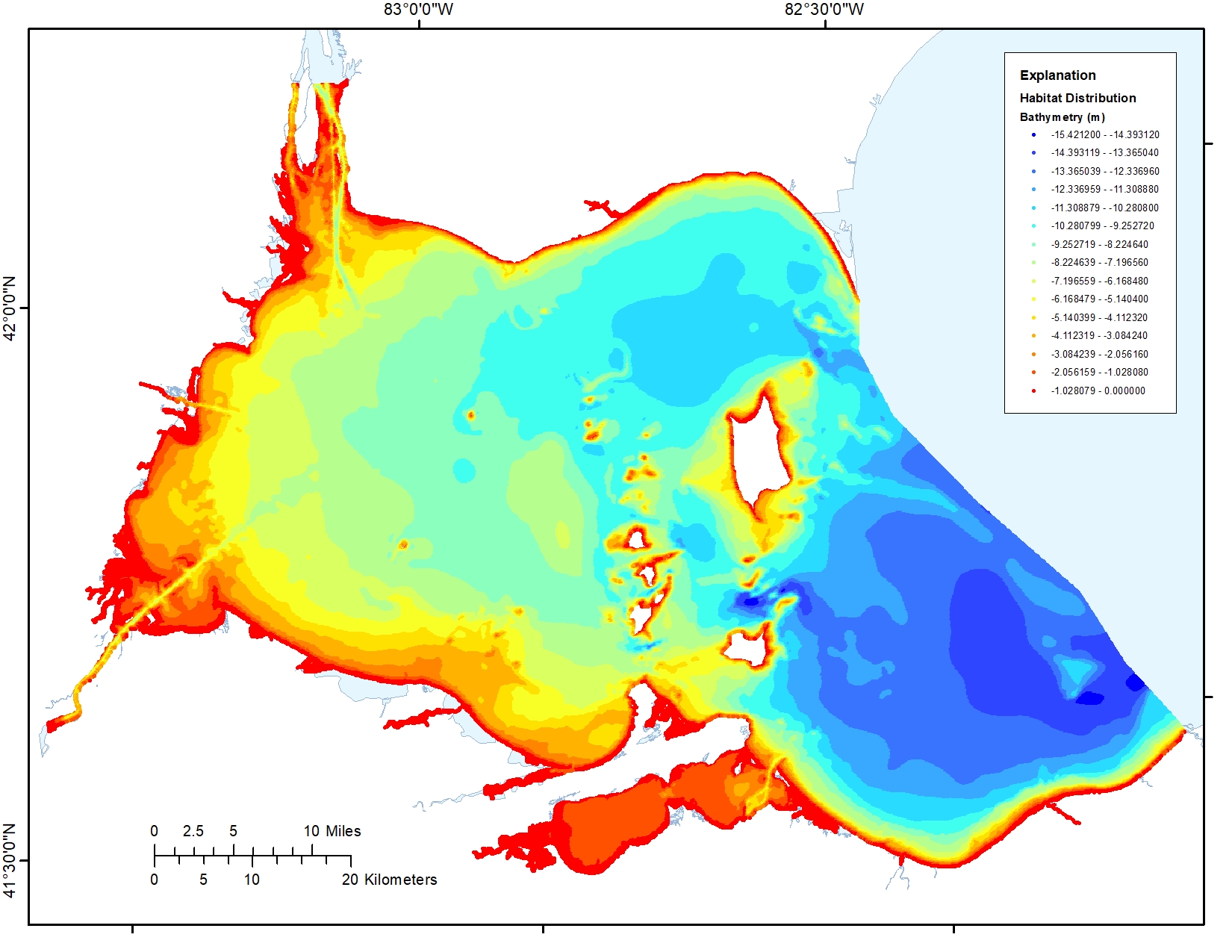


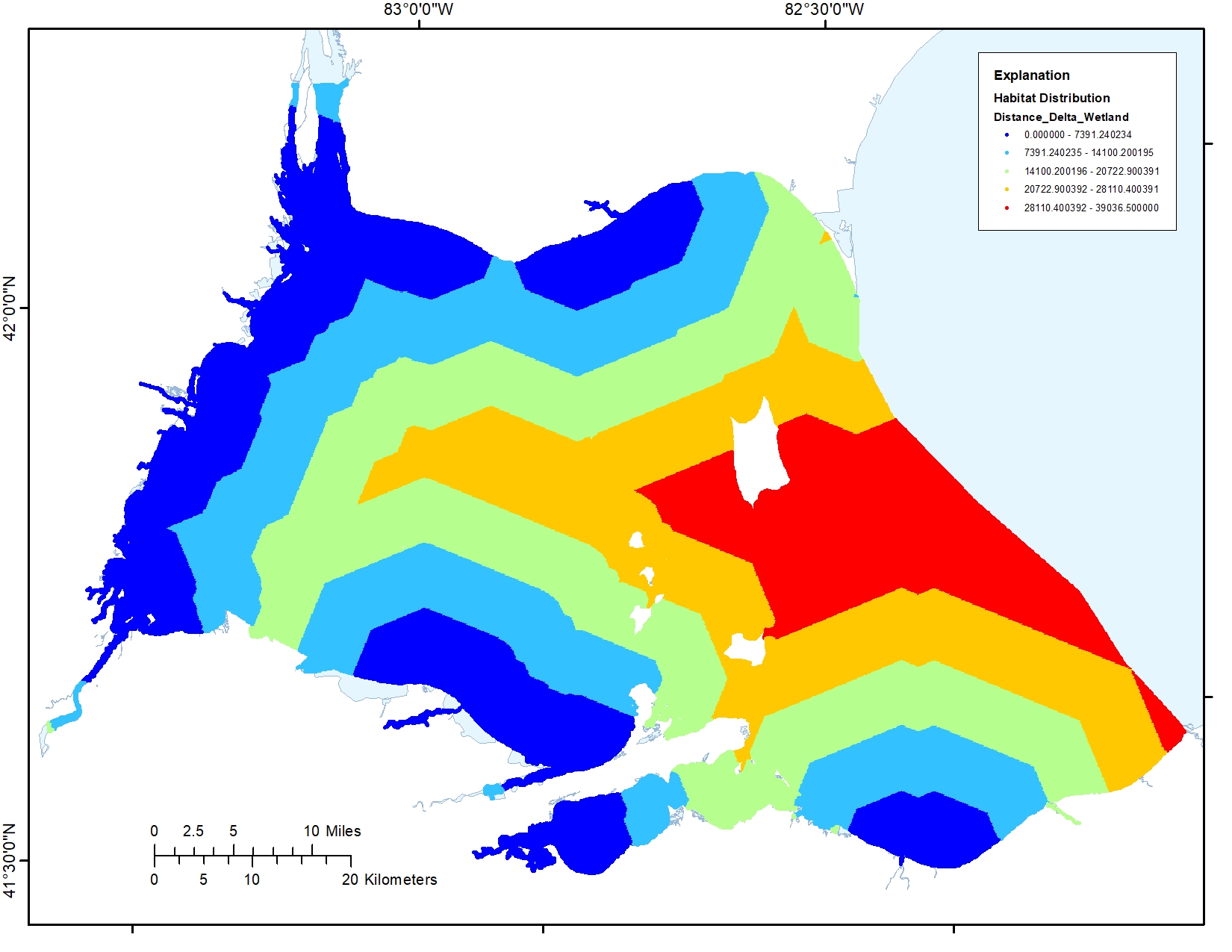


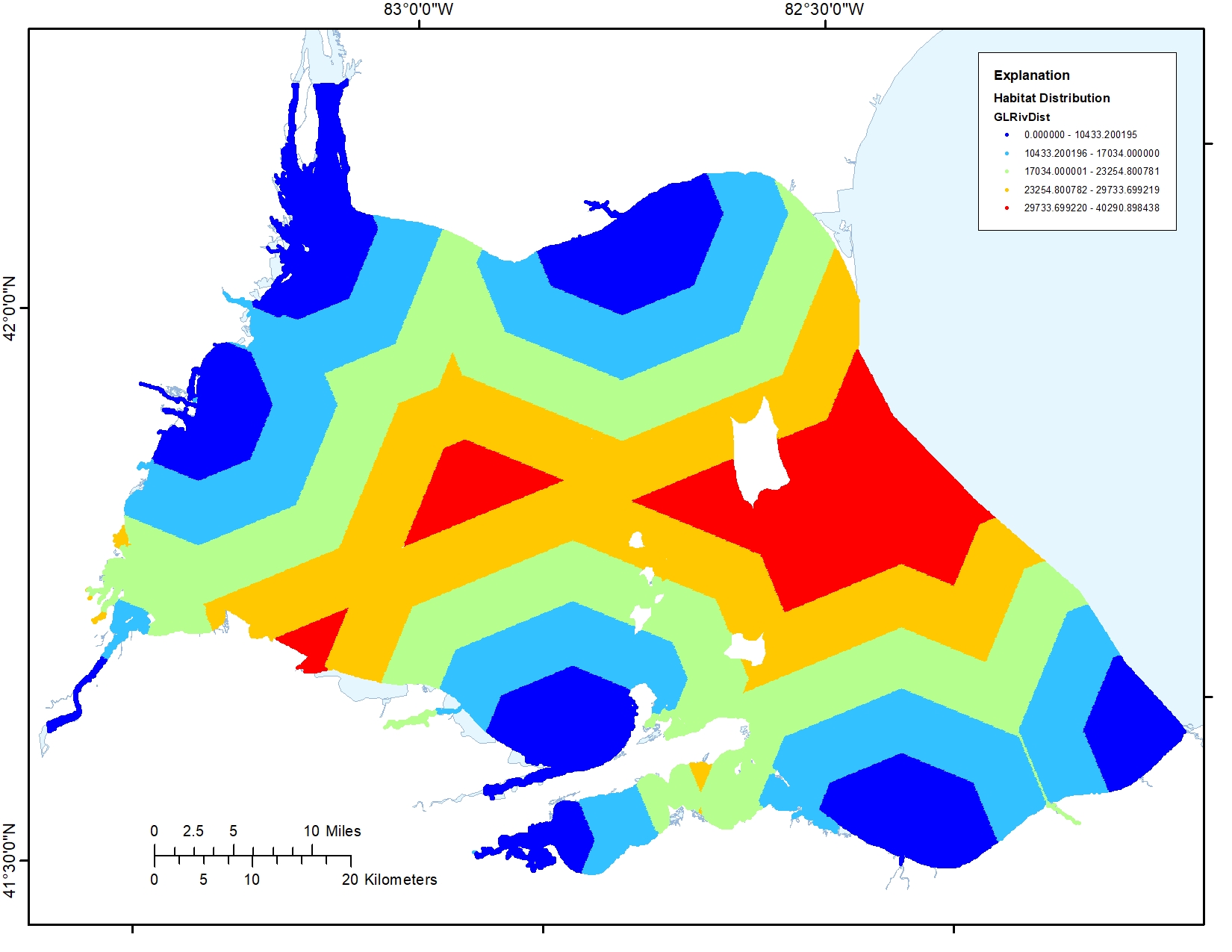


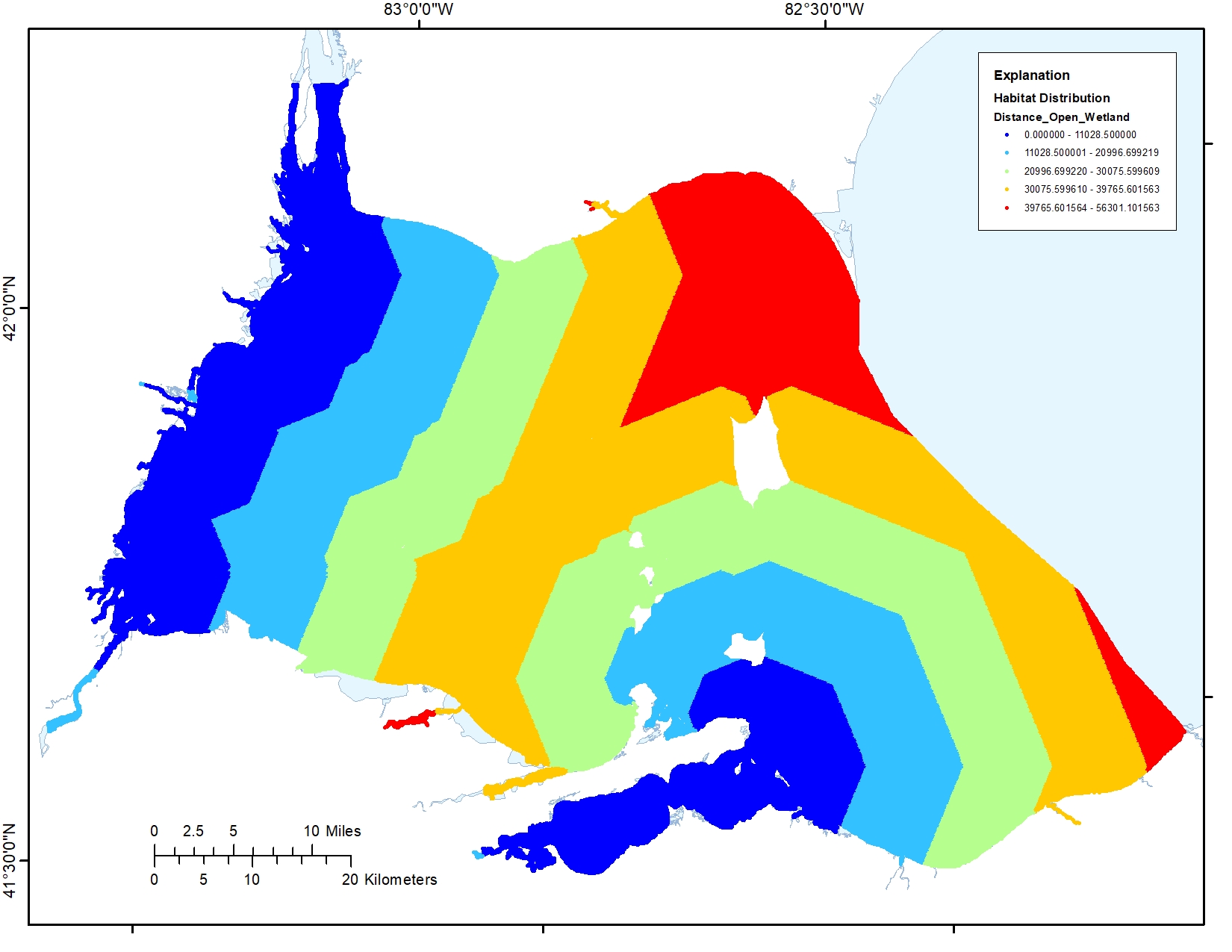


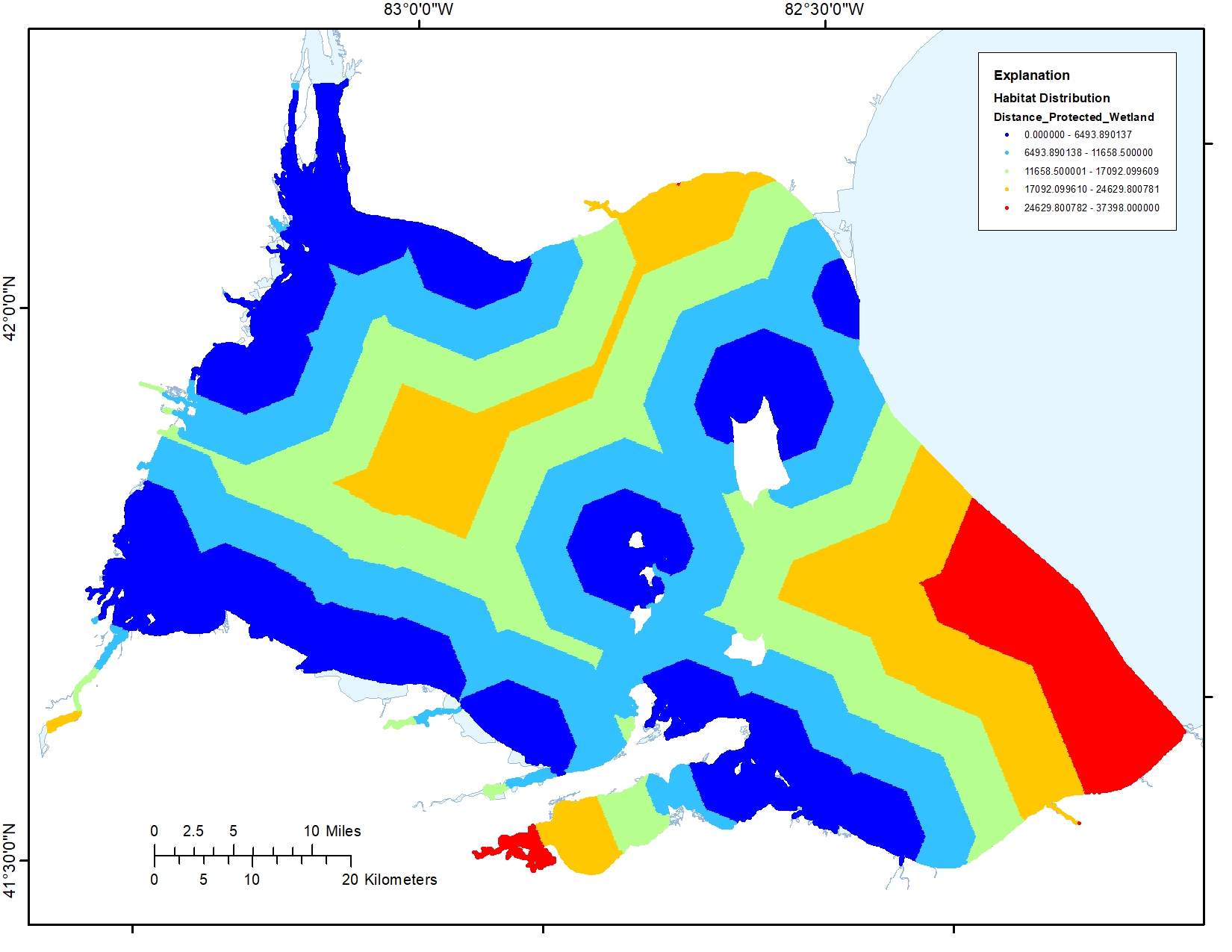


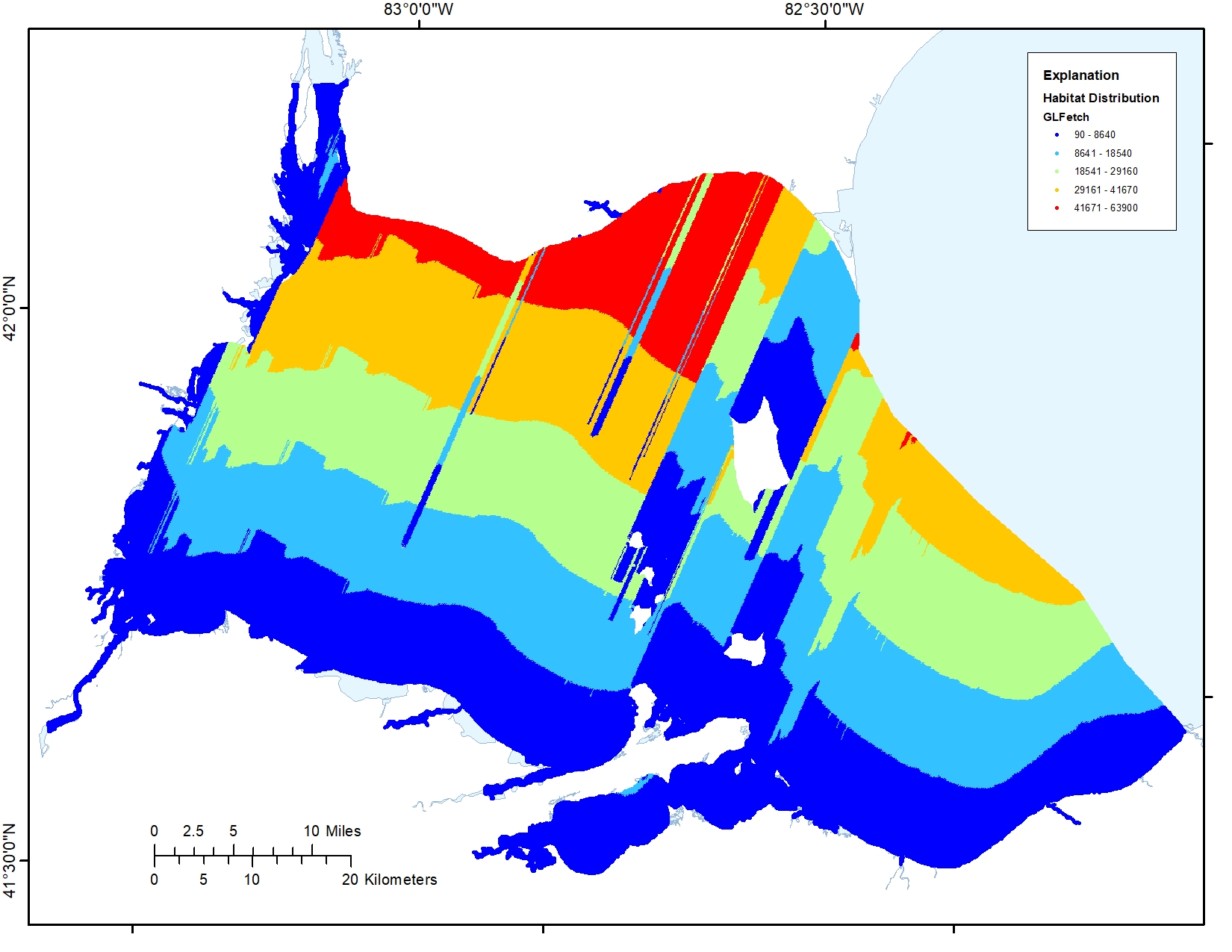


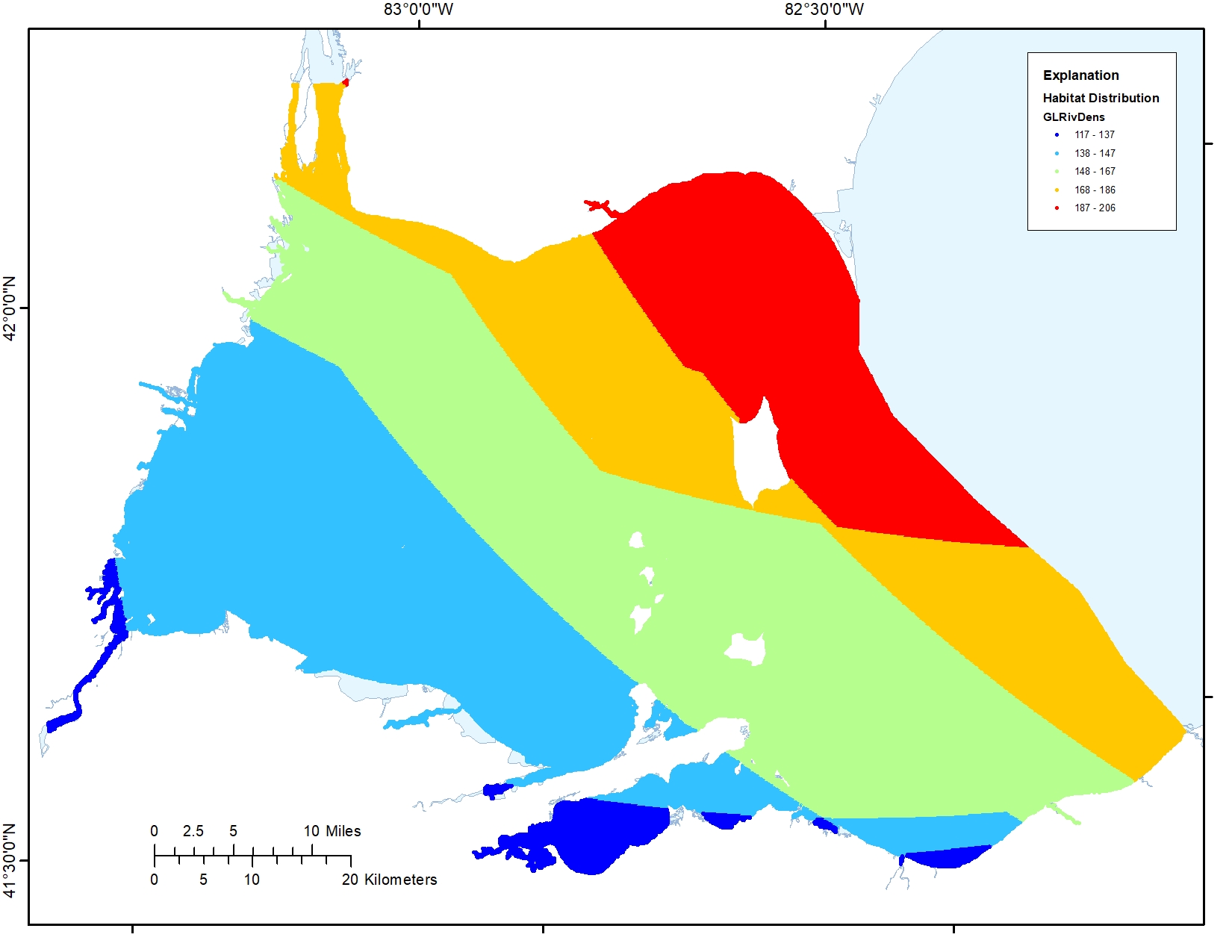


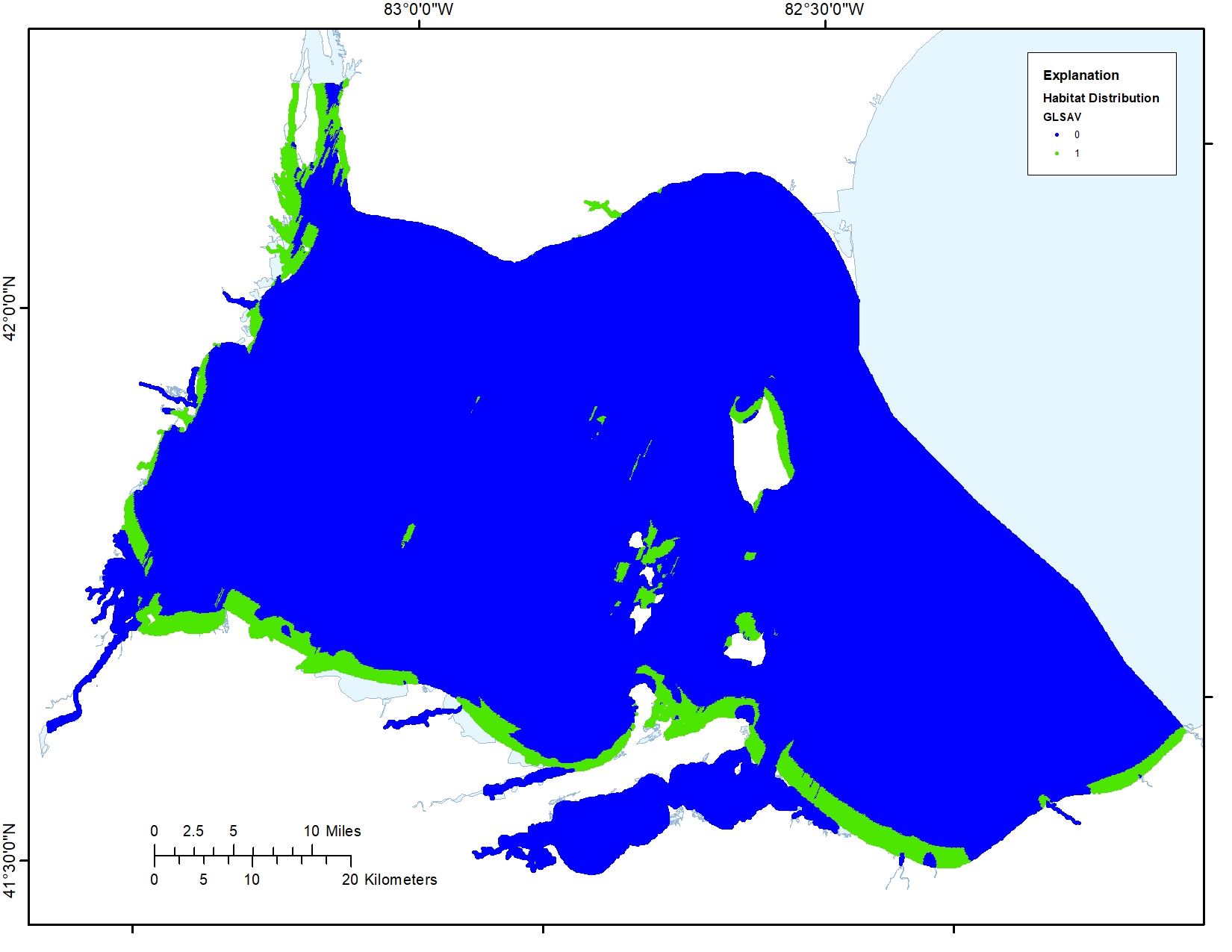


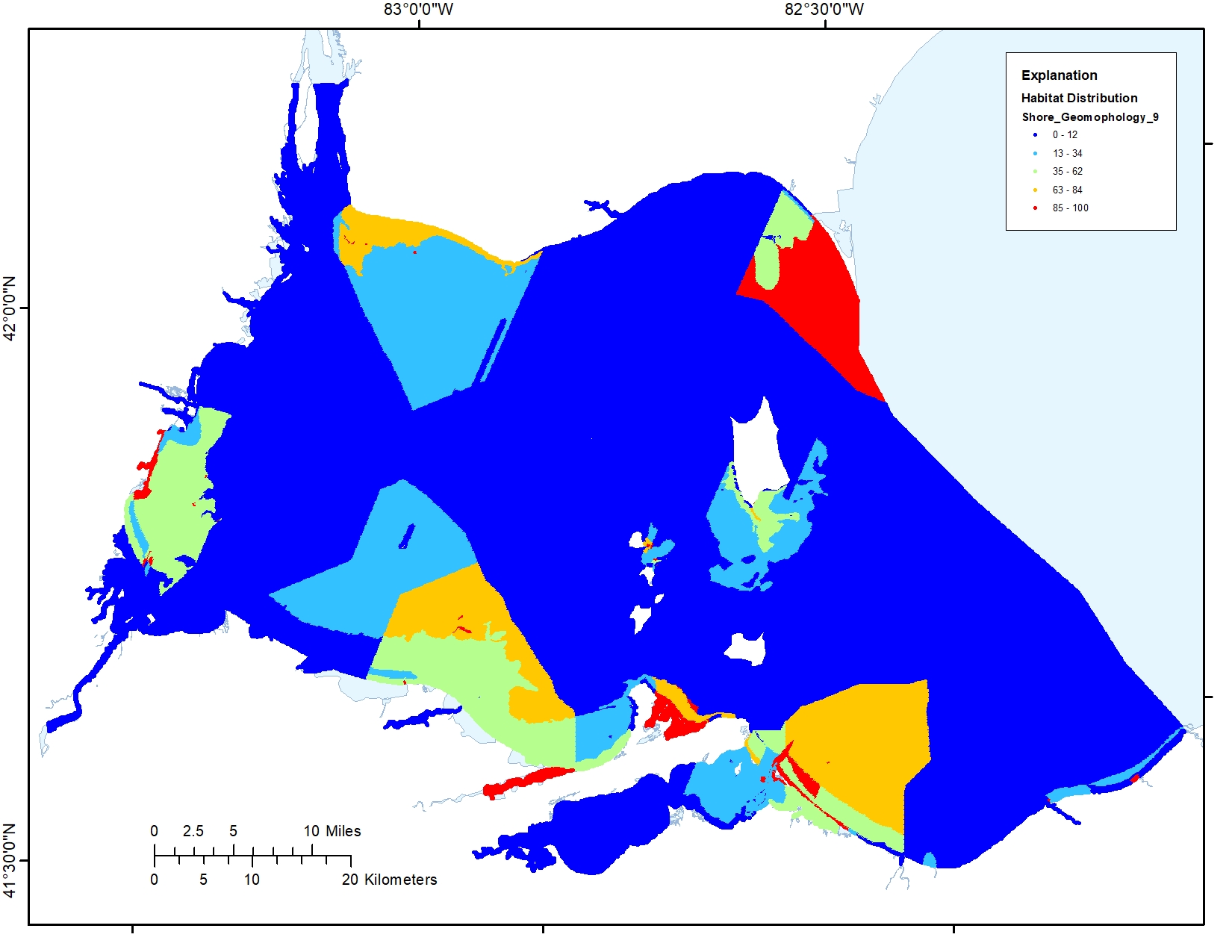


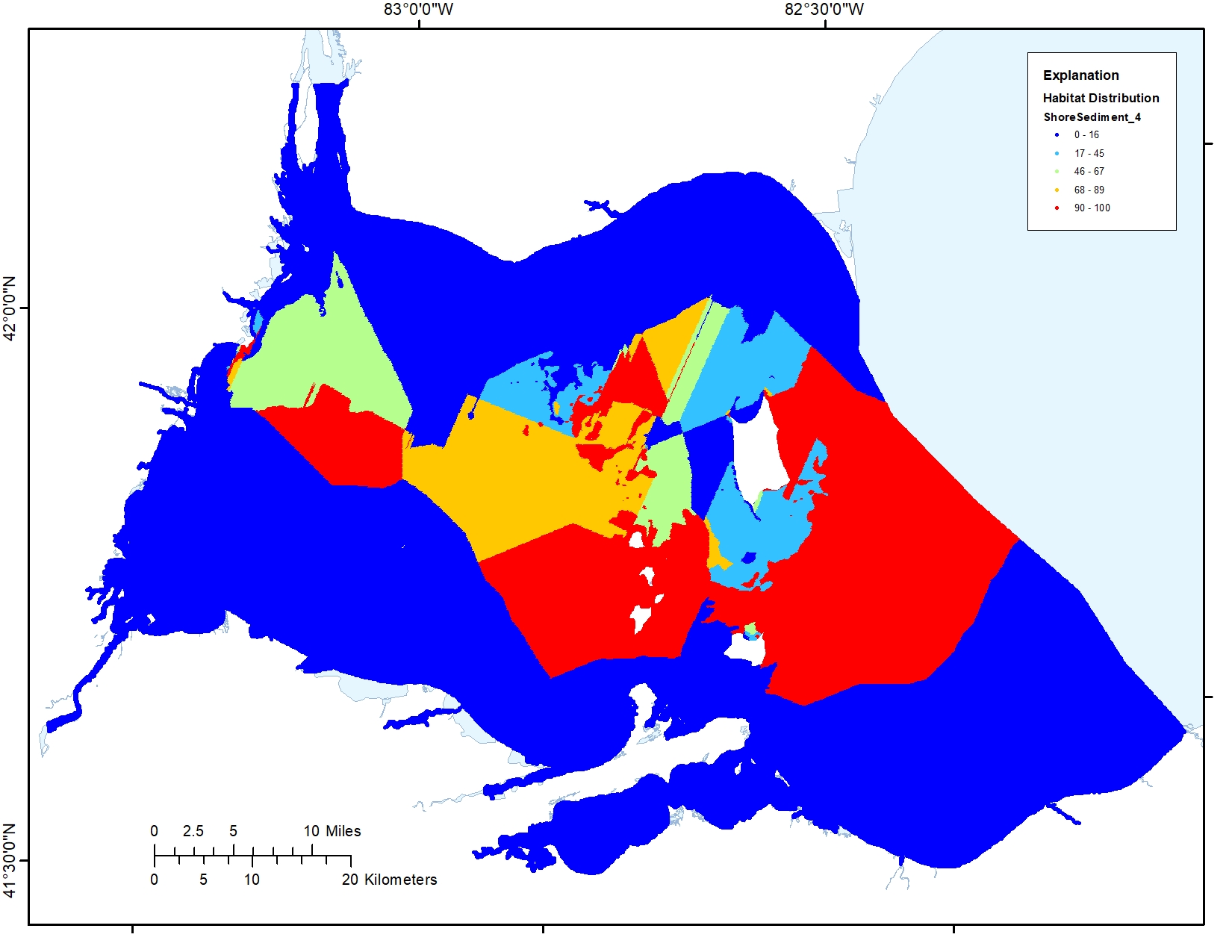


Fig. S2. a – i. Western Lake Erie maps of values of variables used in the Silver Chub Disturbance model in addition to those of the Potential model.


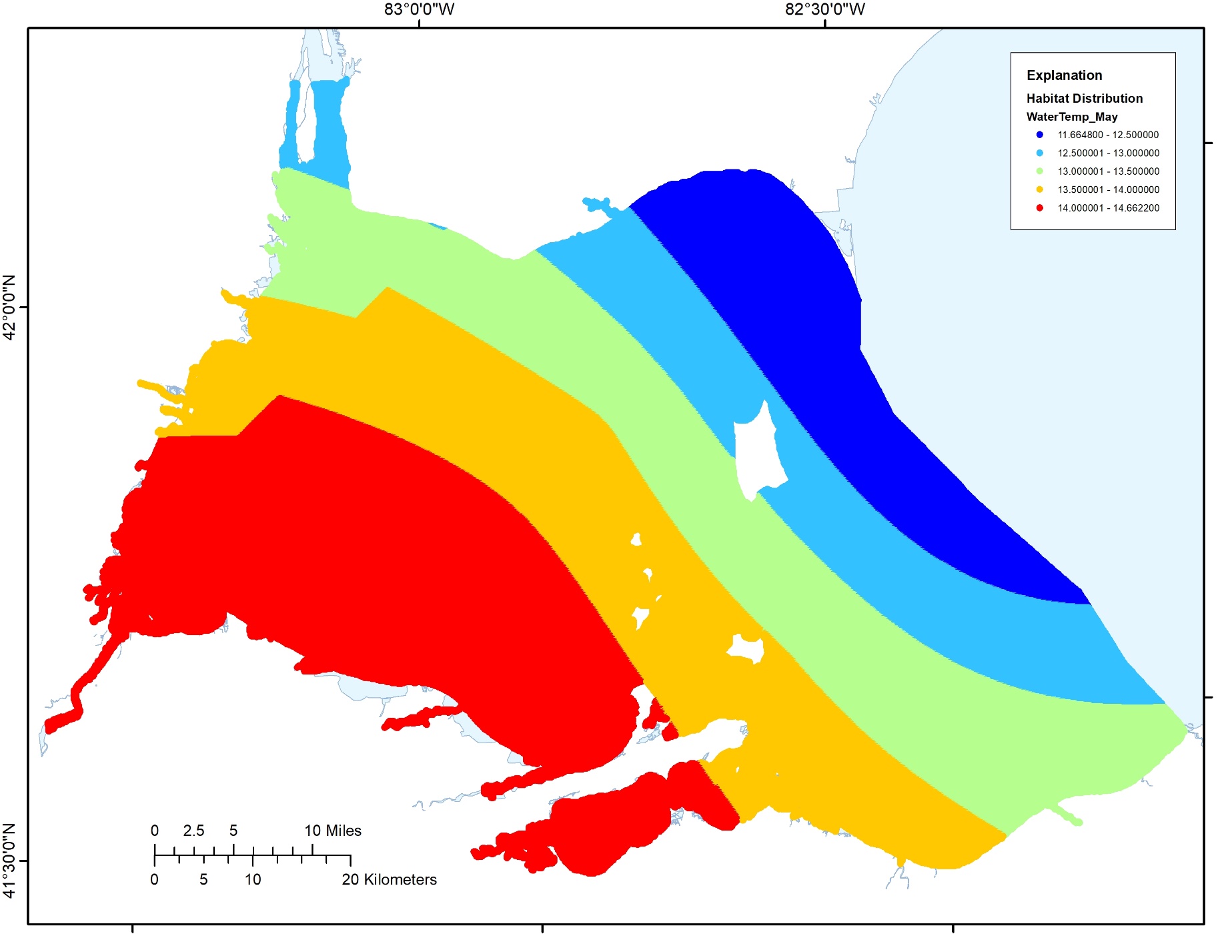


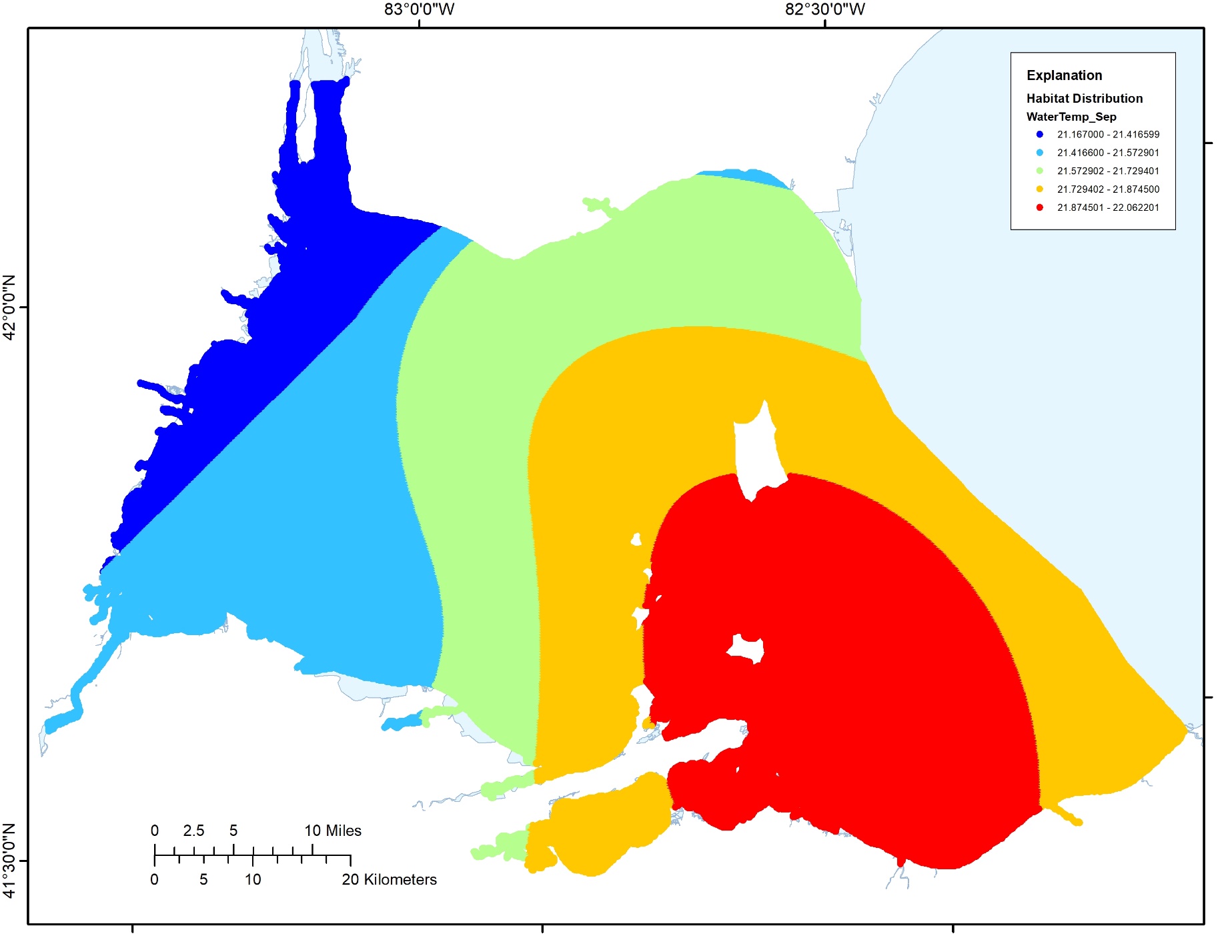


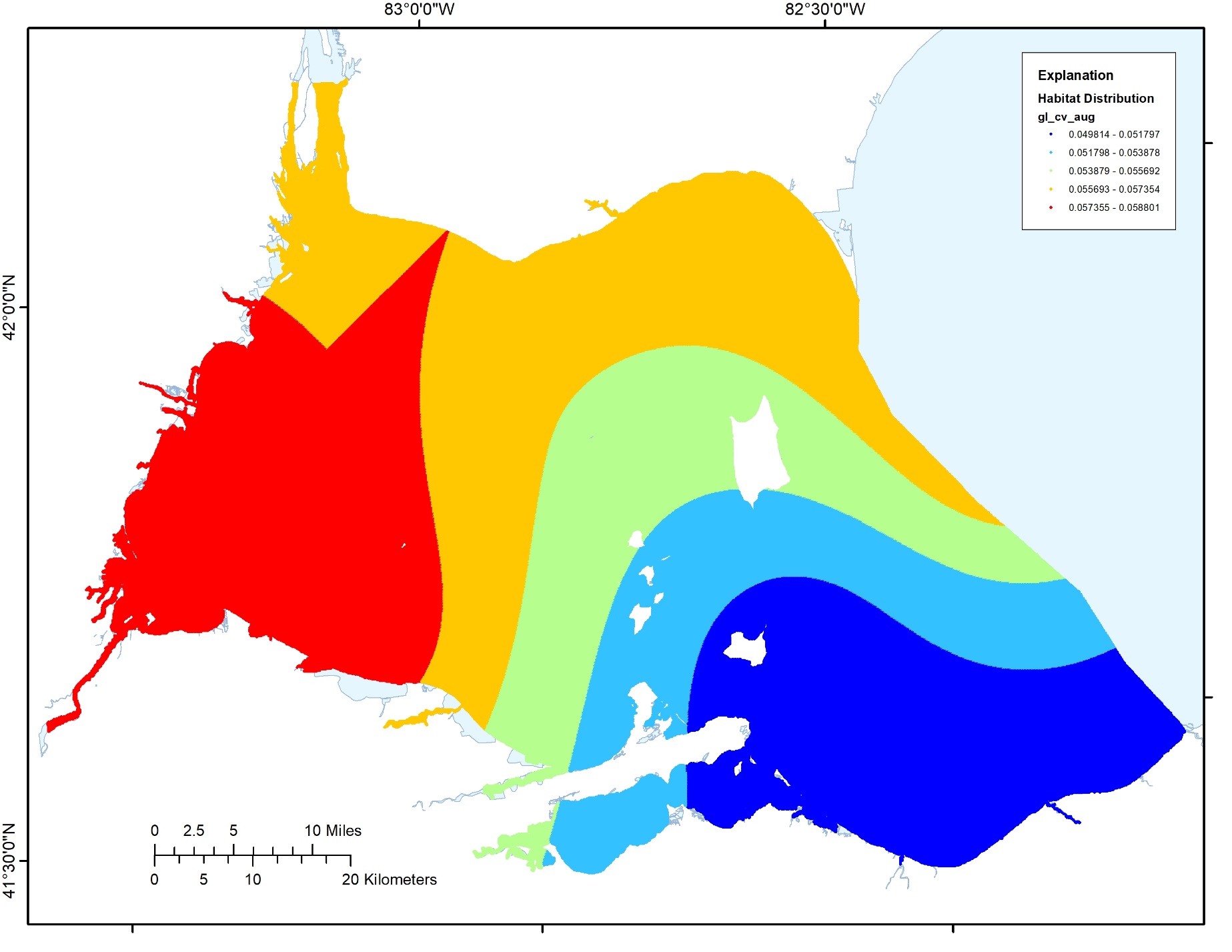


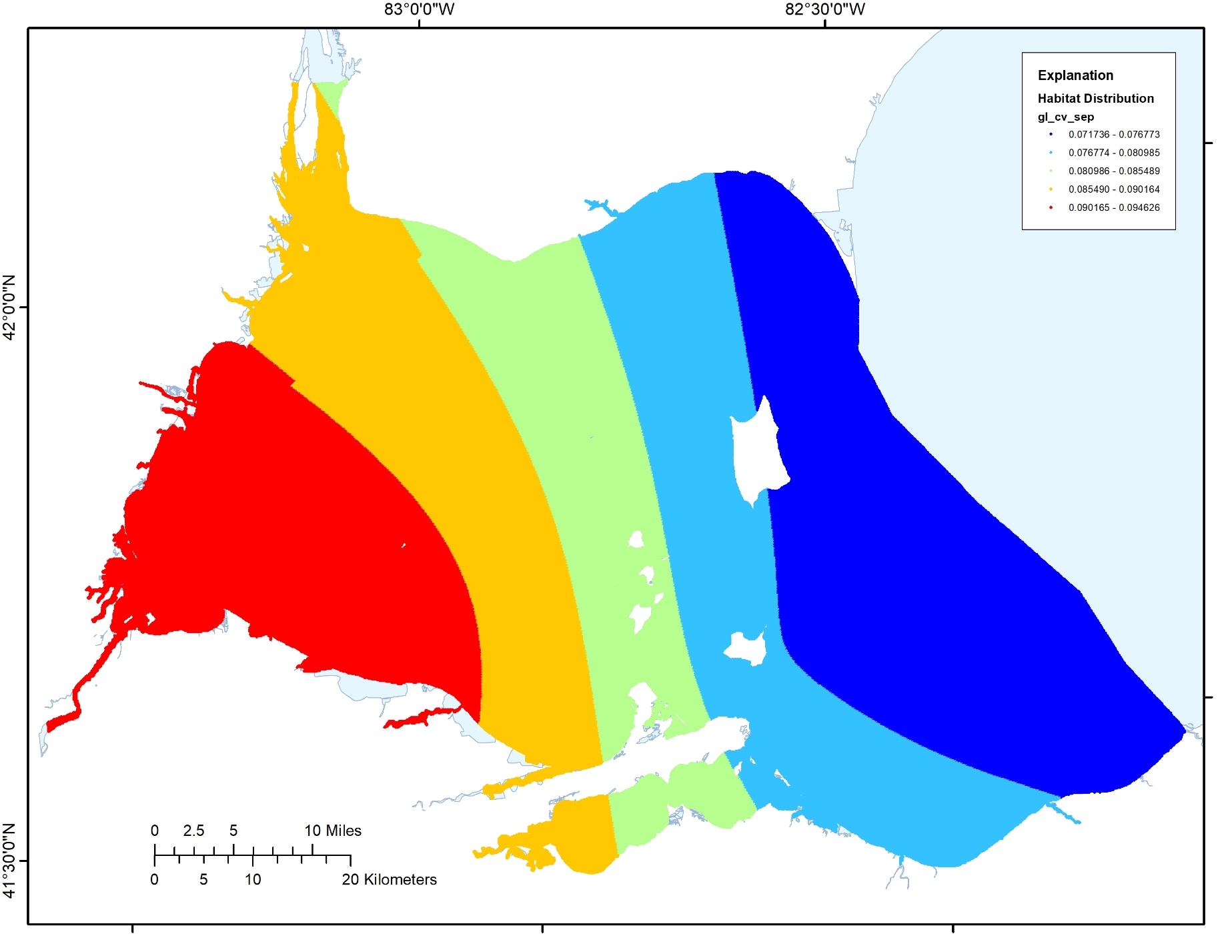


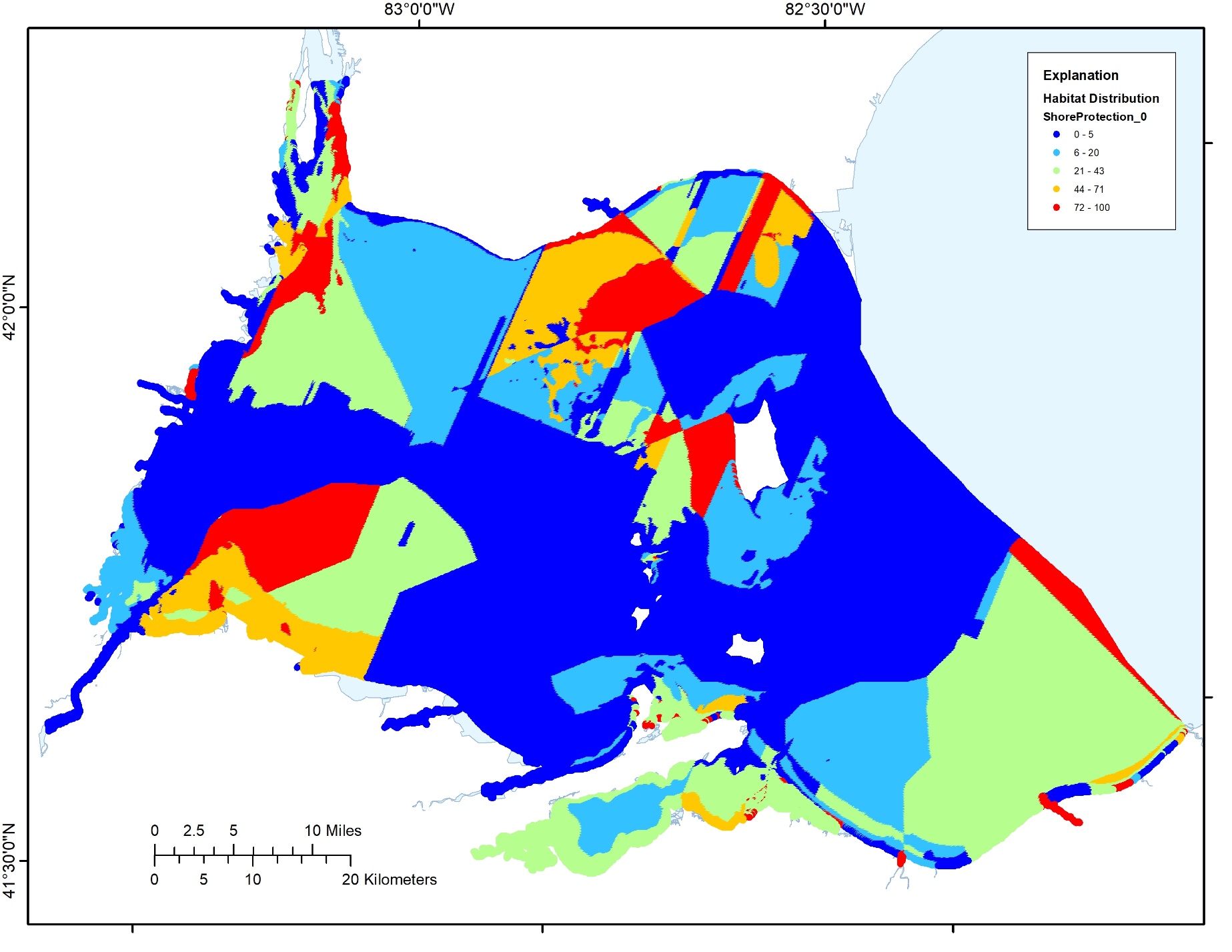


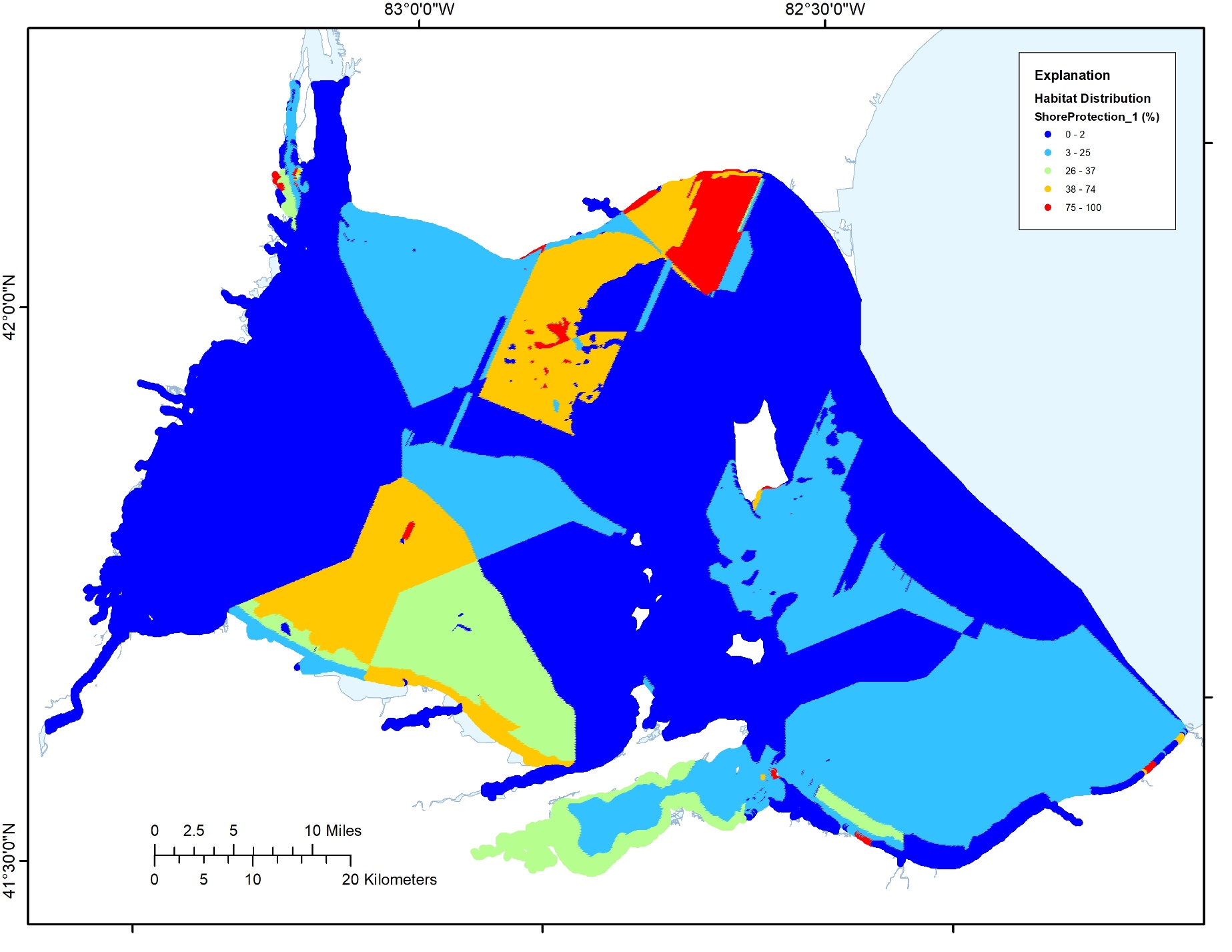


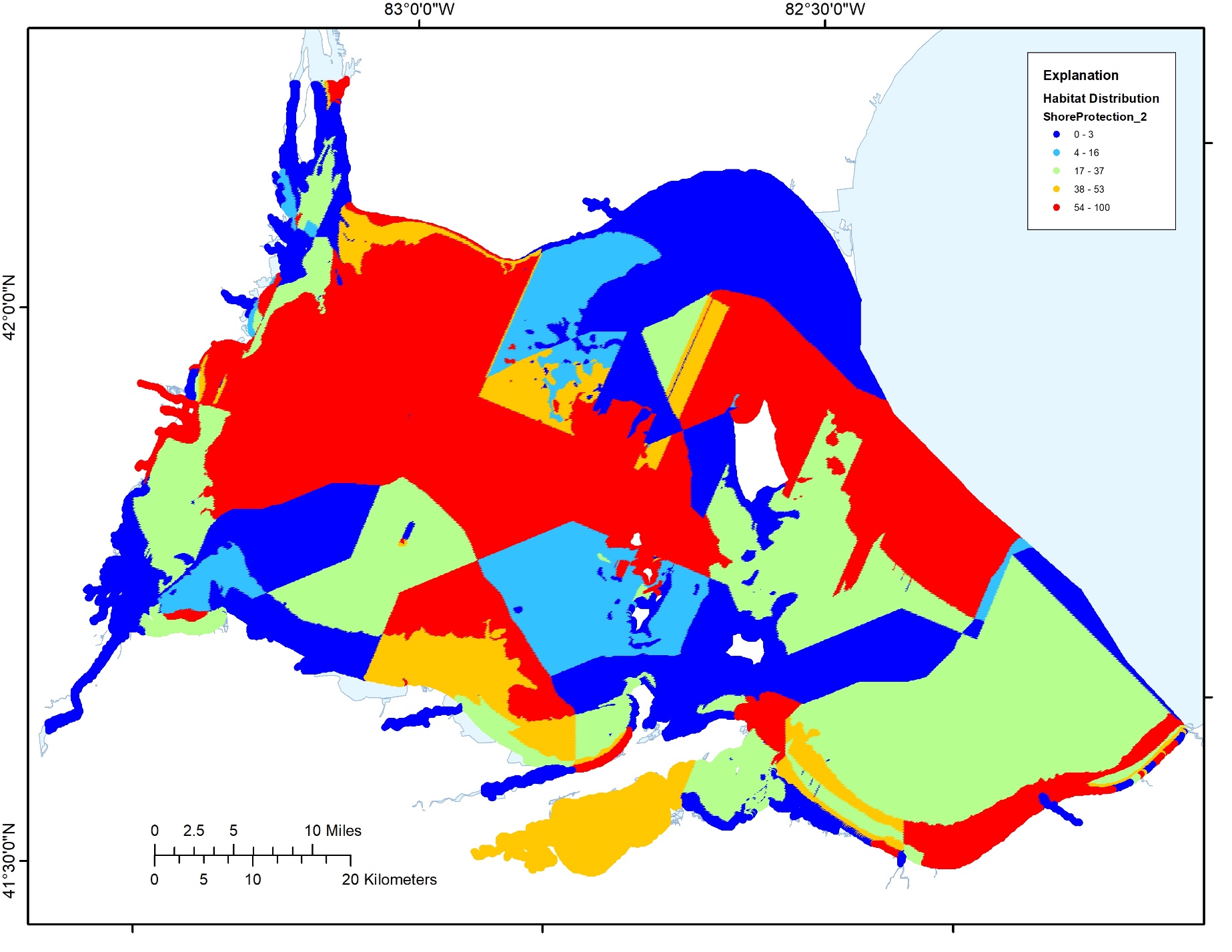


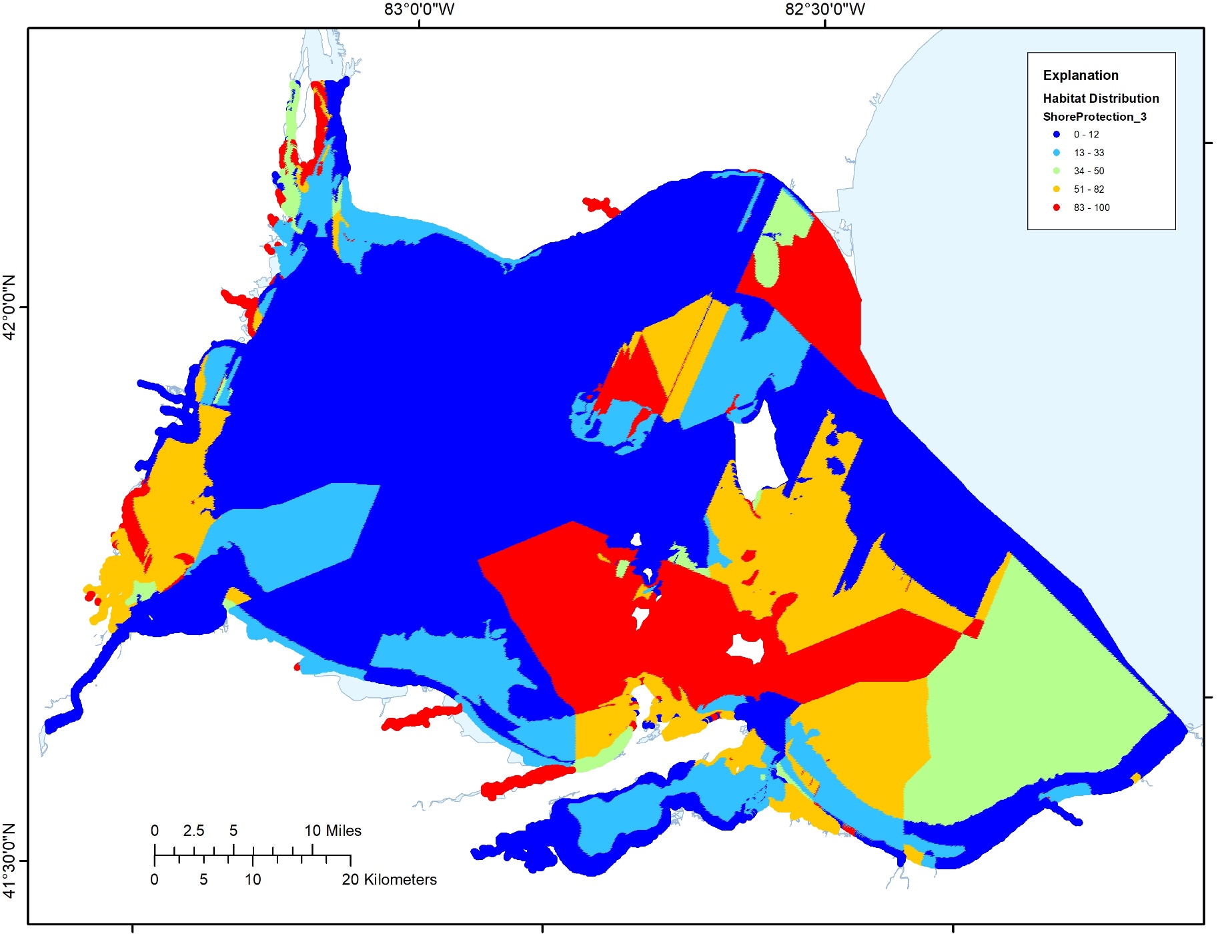


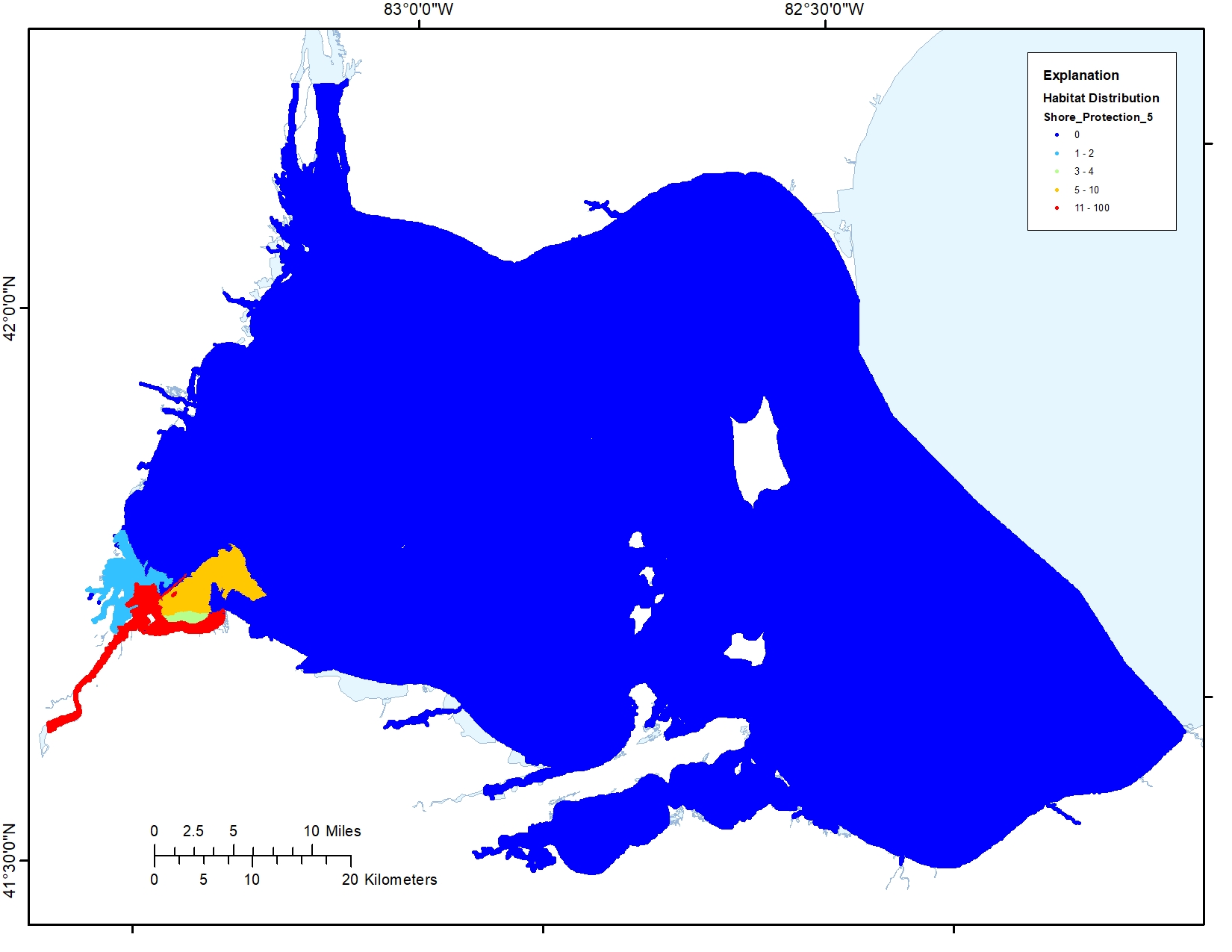


Figures

Fig. S3?? Relative influence weightings for the Potential and Disturbance models based on tracing pathways through each neural network from input variables to prediction neuron. Only the first 10 variables (show from left to right along the abscissa) were used in the Potential model. All 19 variables were used in the Disturbance model. Note the difference in axis scales.


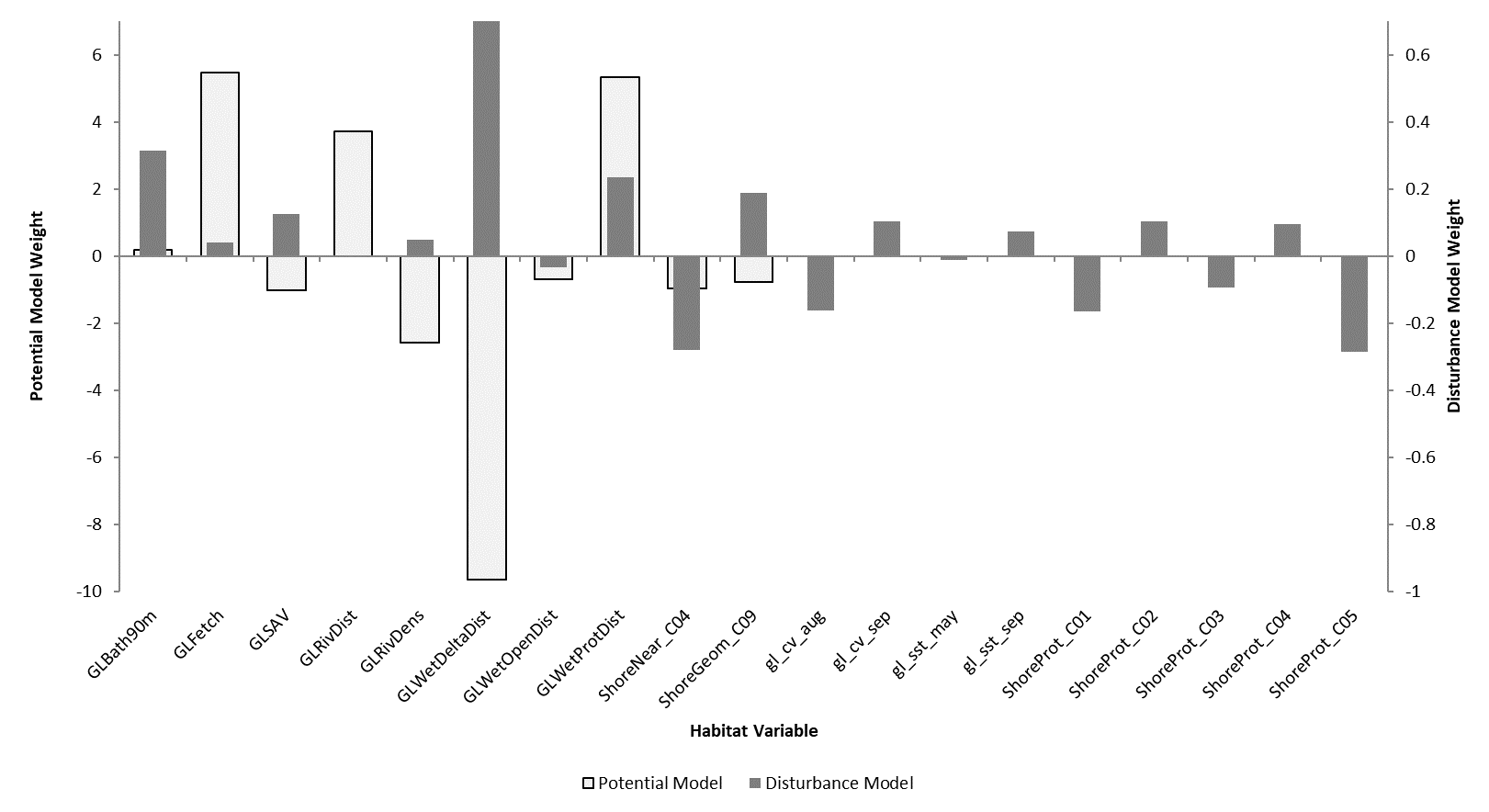

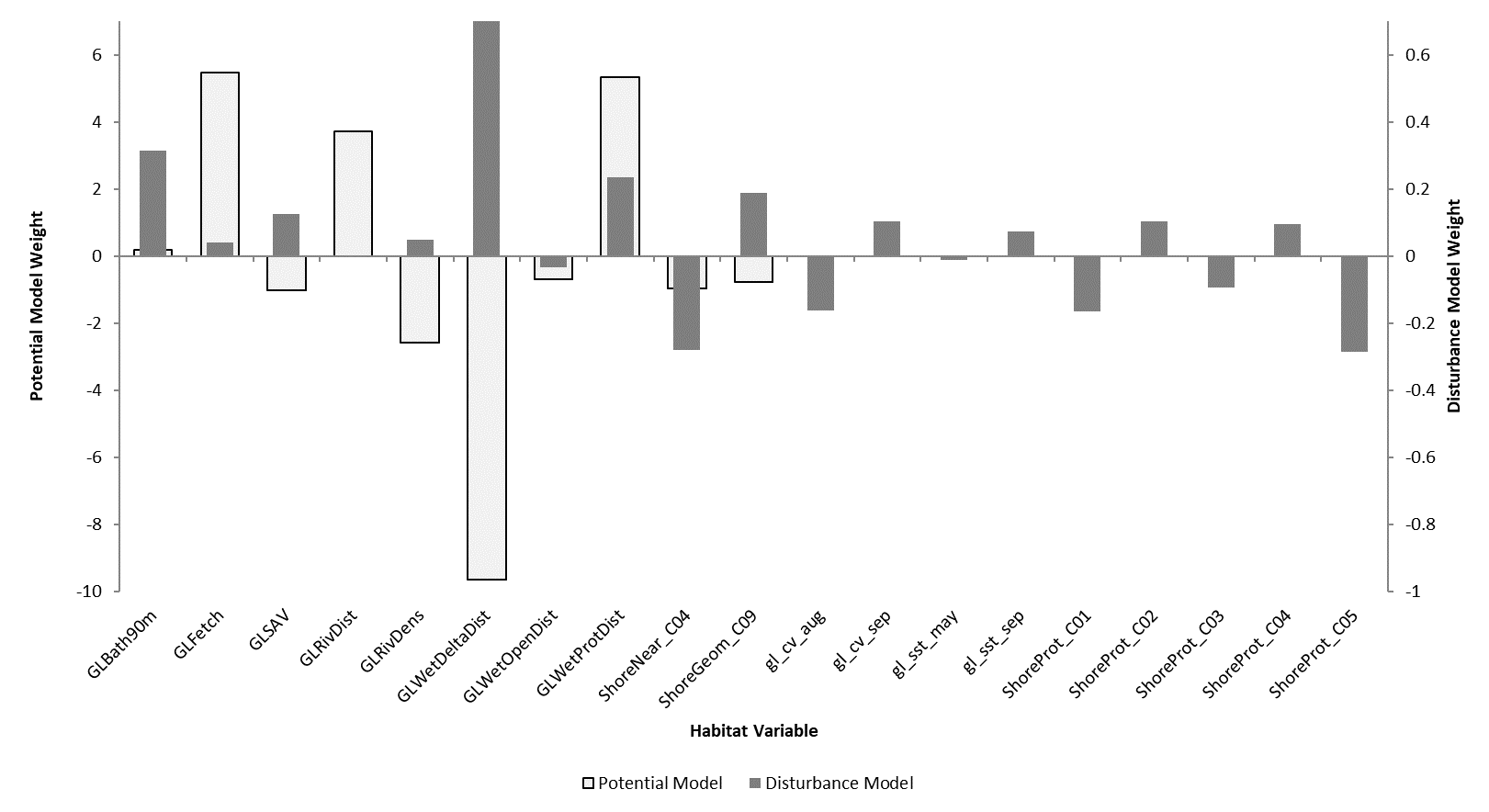


Fig. S4. a. – j. Box plots for each disturbance habitat variable used in the Disturbance model, comparing of data distributions at spatial units that differed (or did not change) from predictions of the Potential to the Disturbance models. Data means are indicated with and “X”. The horizontal line indicates the data median. The upper and lower ends of the boxes are the upper and lower quartiles, respectively. Ends of the whiskers indicate upper and lower 95% confidence interval. More extreme data are shown by circle beyond the ends of the whiskers. Classes of difference from the Potential Model to the Disturbance model are color-coded and include, Absent to Appropriate (dark green), Absent to Marginal (light green), Appropriate to Absent (red), Appropriate to Marginal (orange), Marginal to absent (yellow), and Marginal to Appropriate (medium green), and No Change (gray).


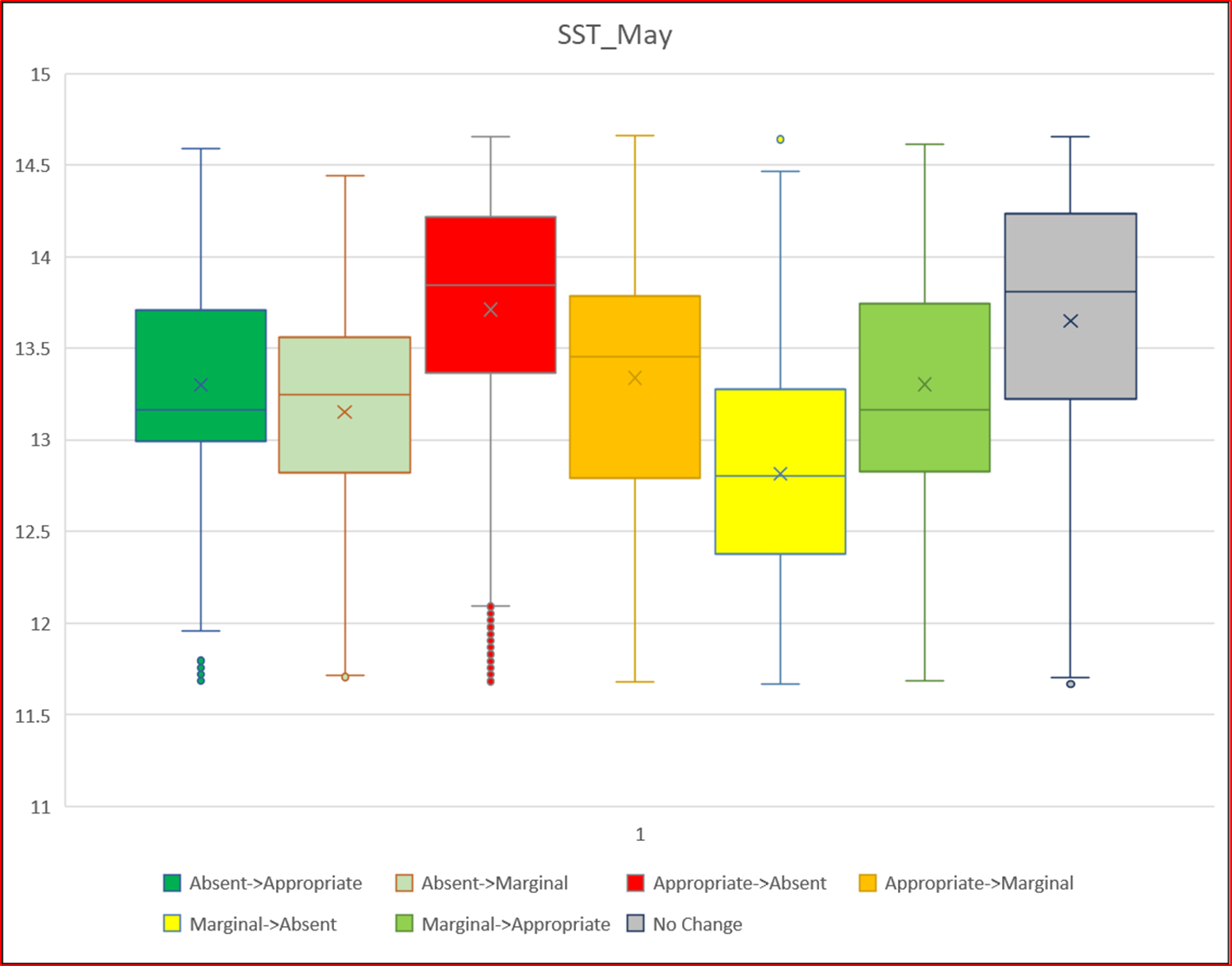


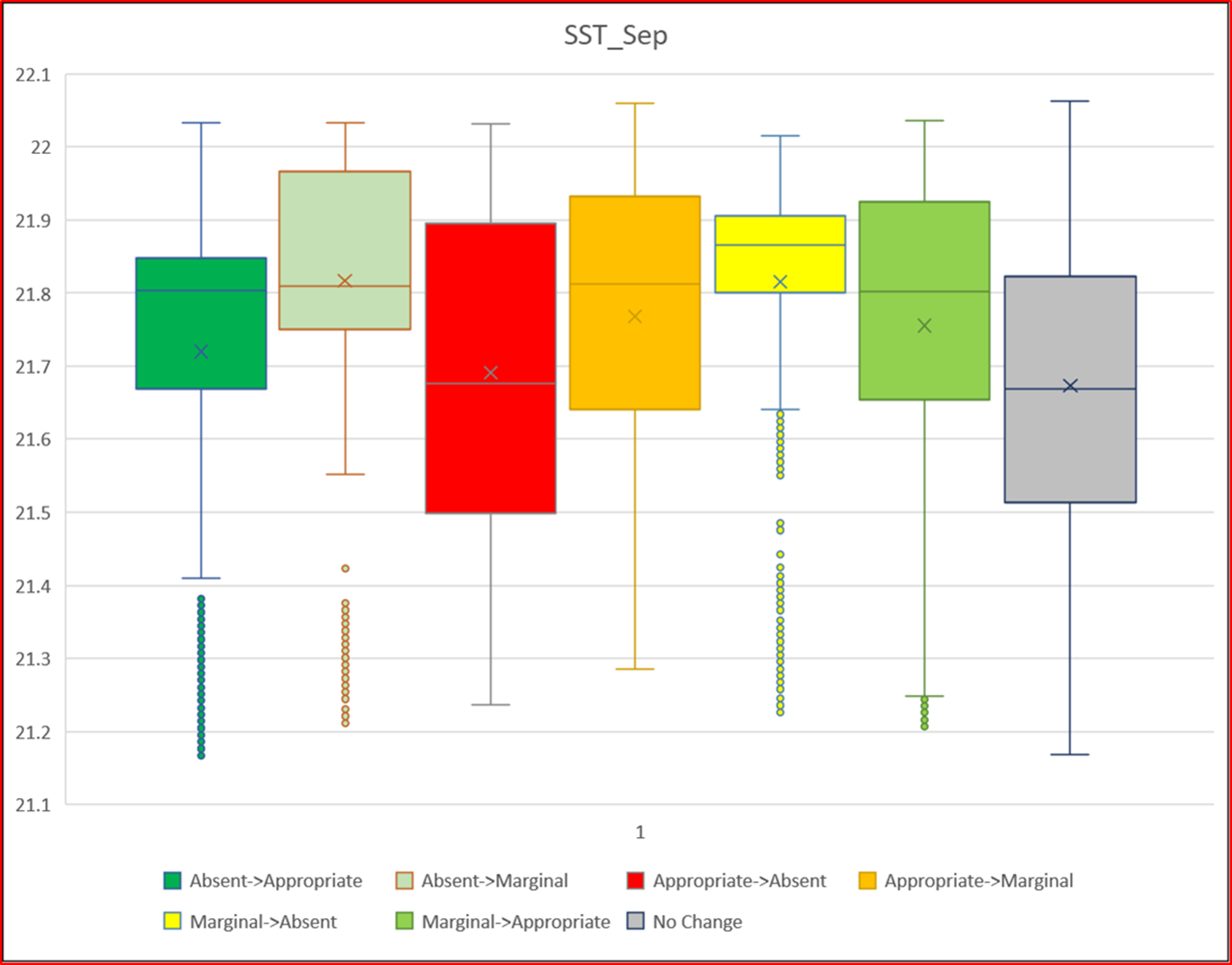


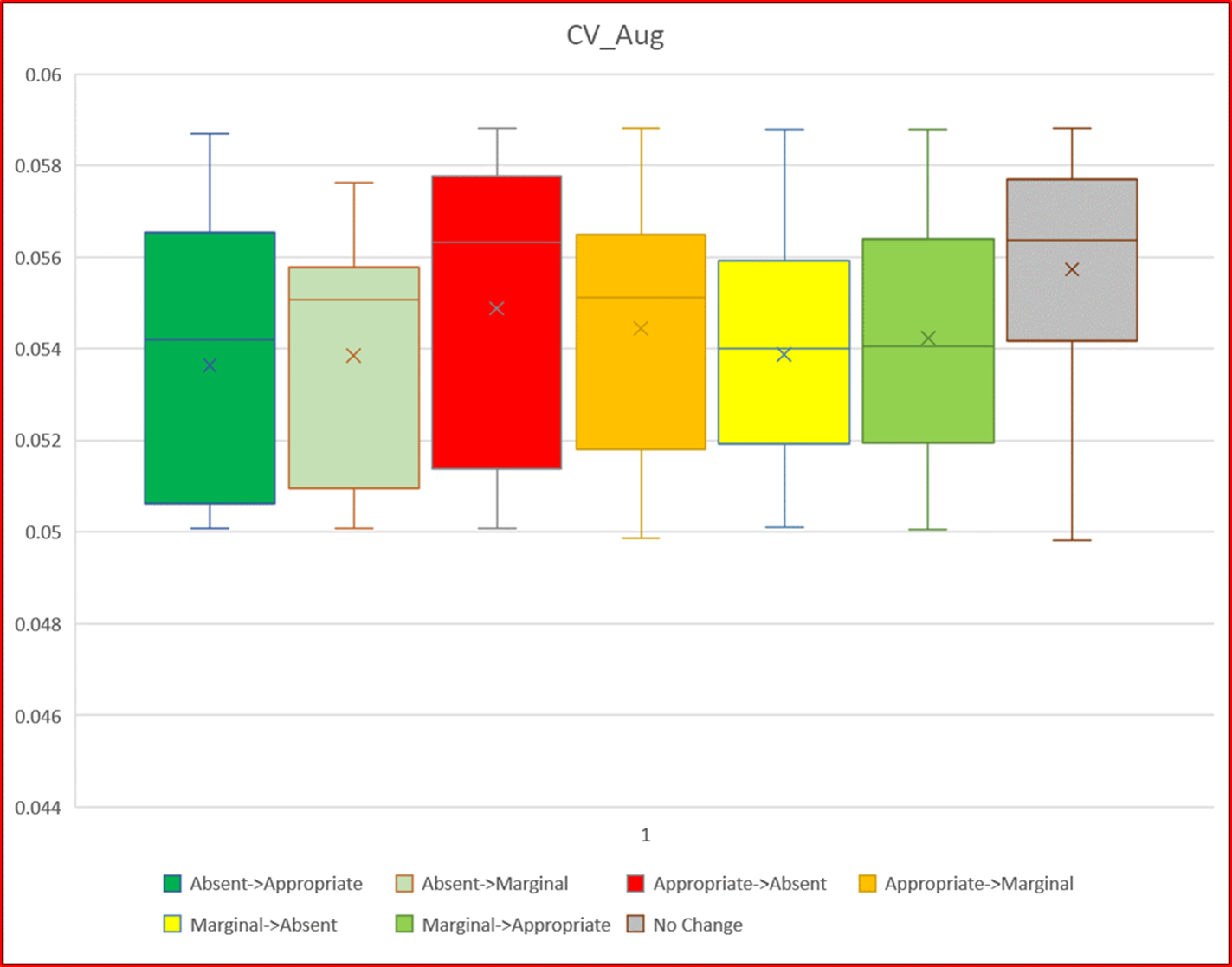


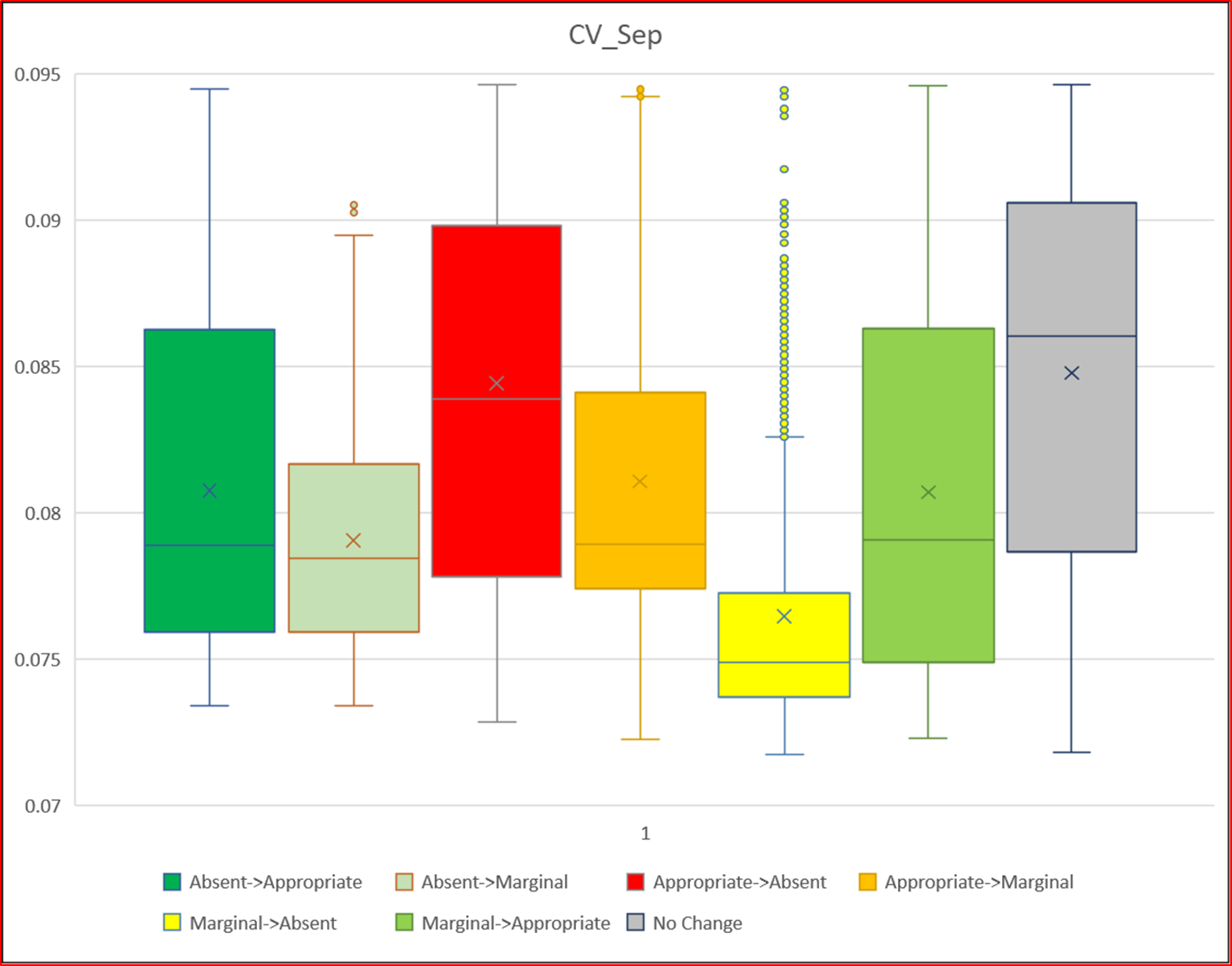


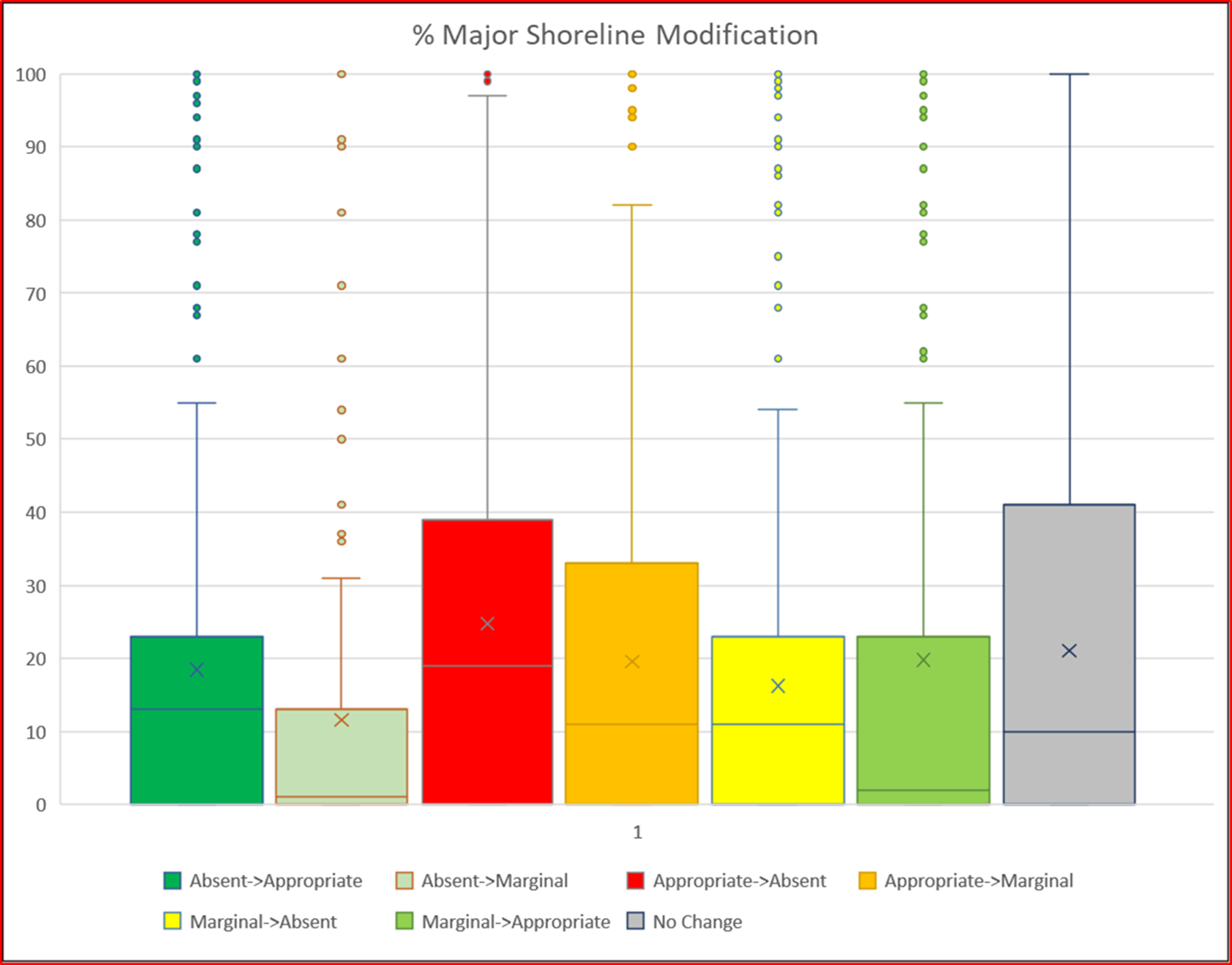


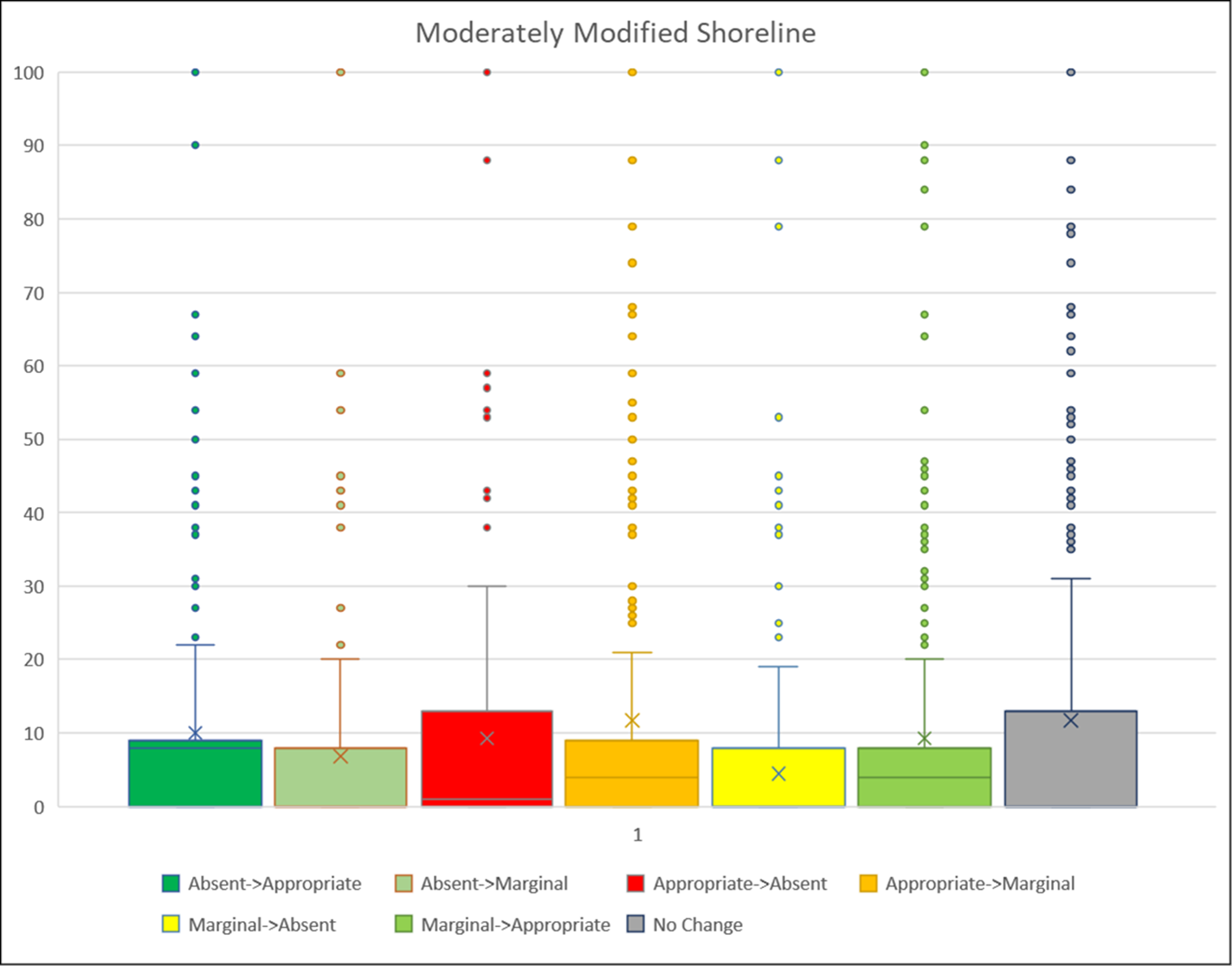


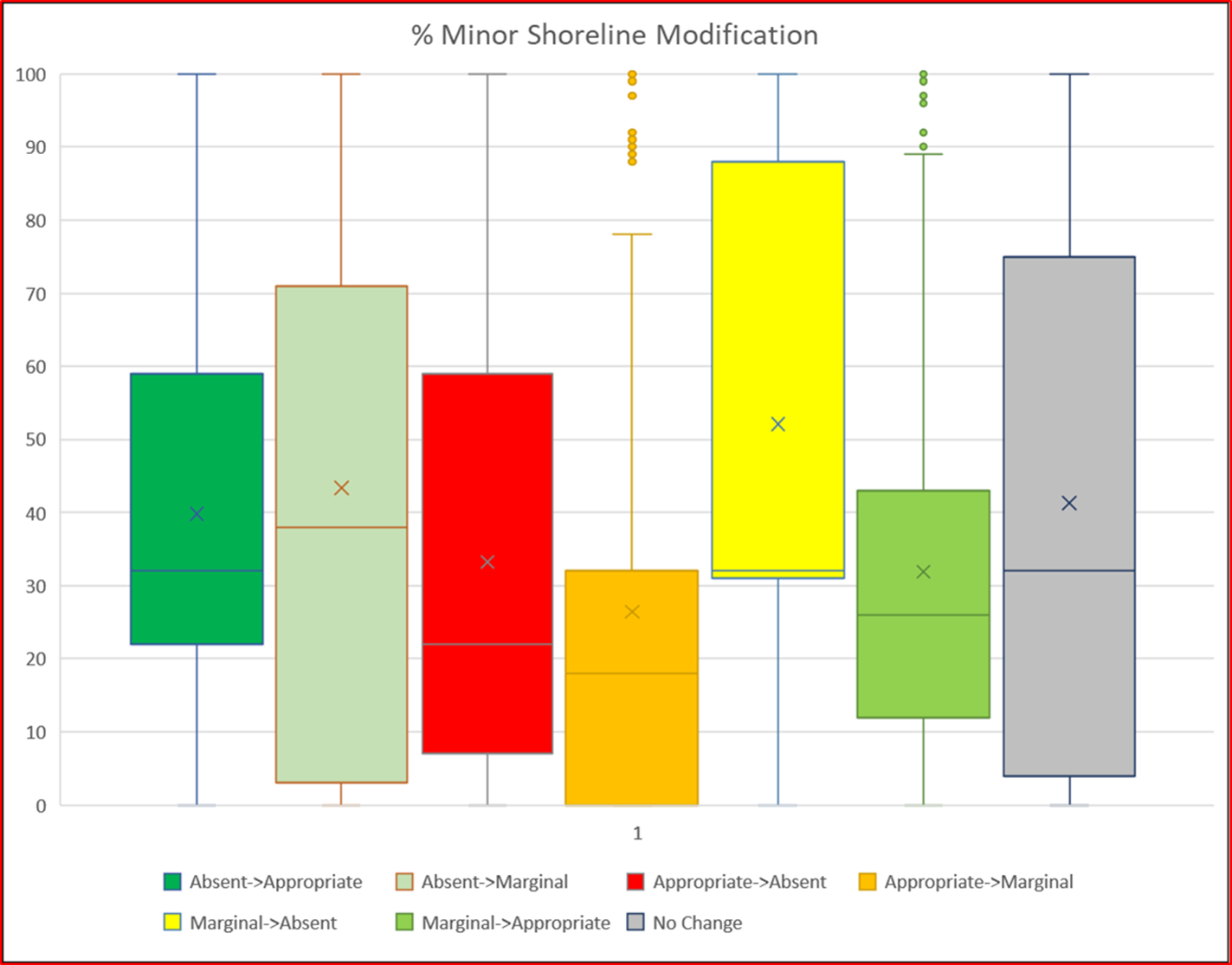


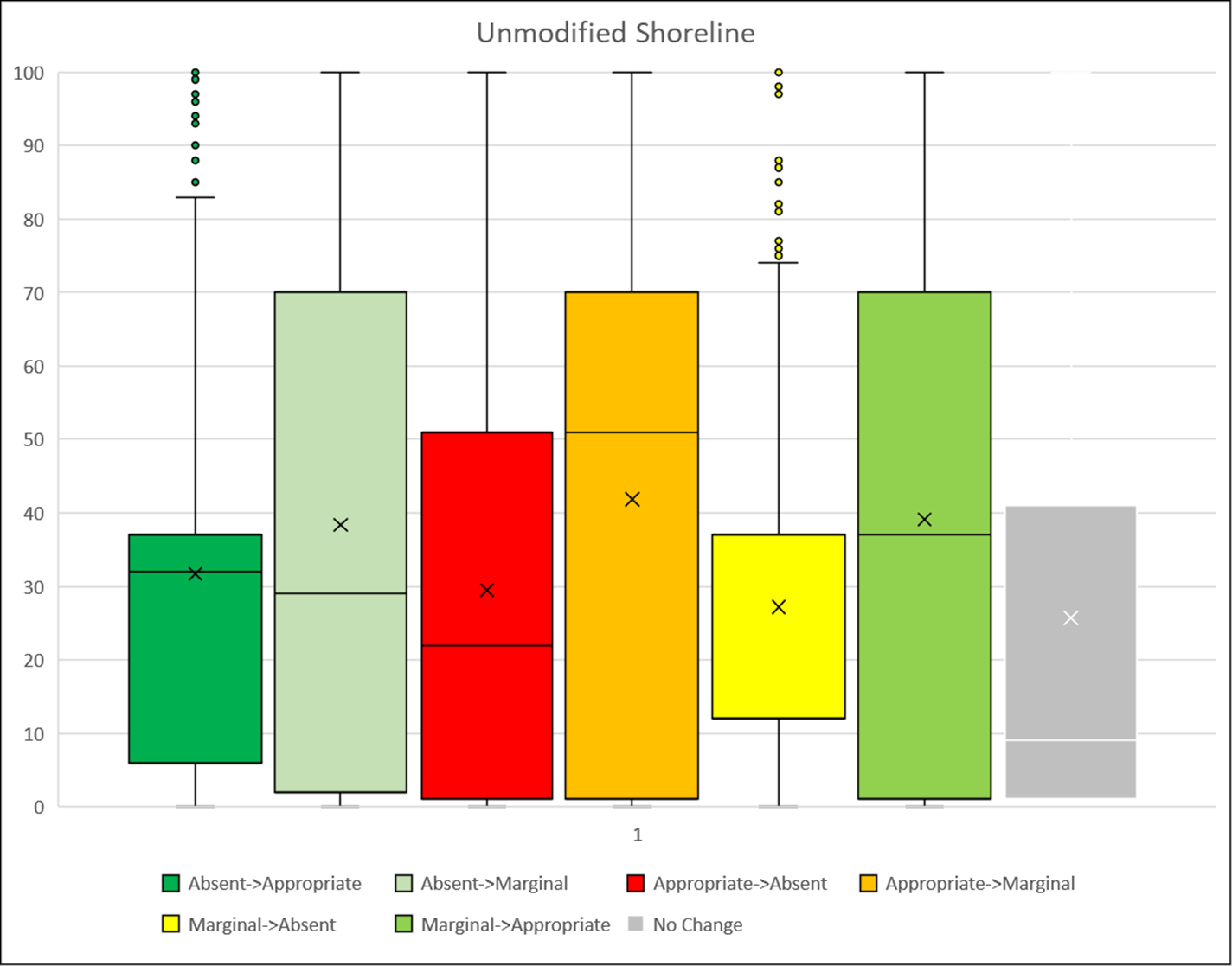


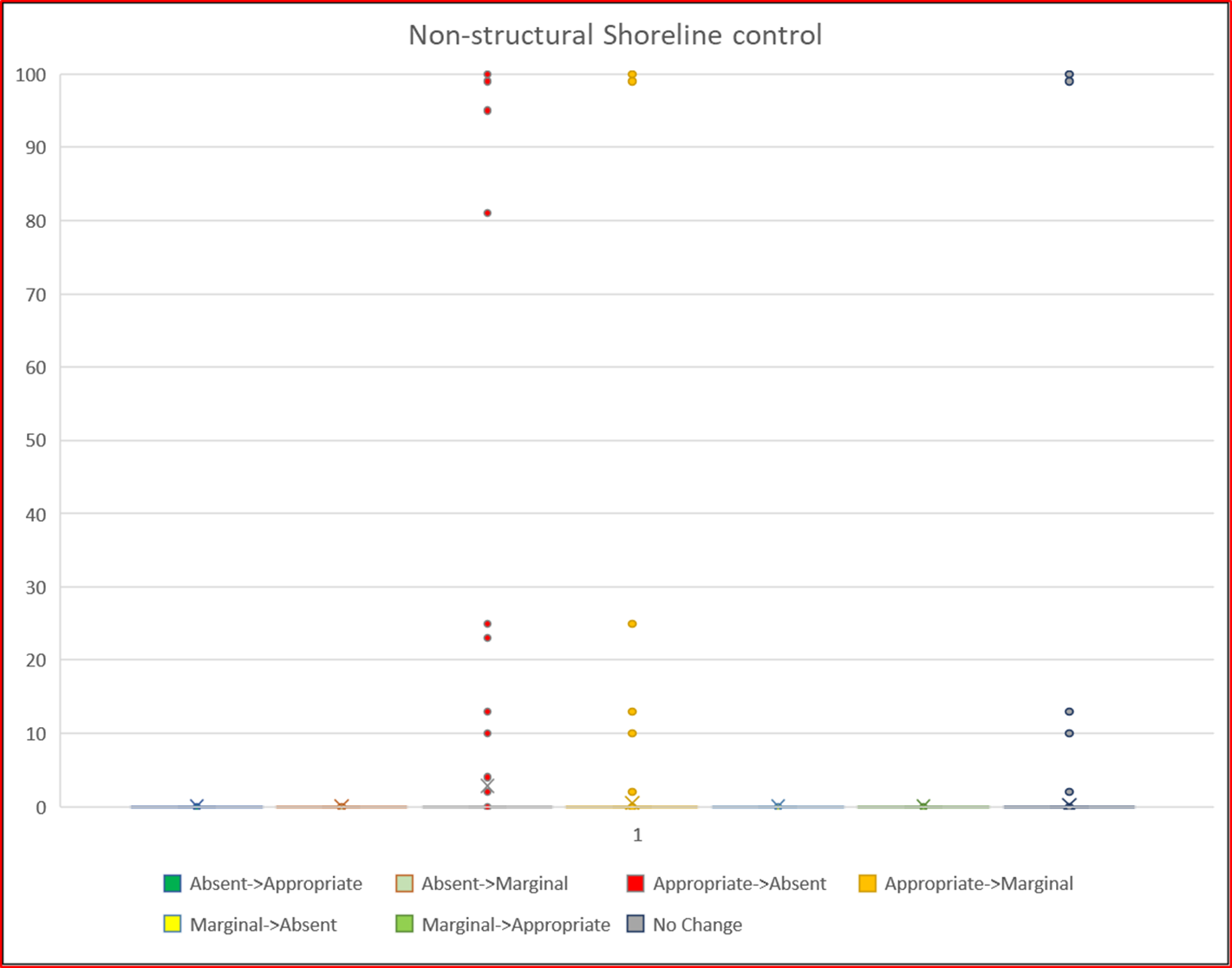

Supplement: Supplementary file 1 — Supplementary Material [file ECE3-10-12076-s001.docx]
