# Supplementary material for: Habitat characterization and species distribution model of the only large‐lake population of the endangered Silver Chub (Macrhybopsis storeriana, Kirtland 1844)
Source: Ecol Evol. 2020 Oct 7;10(21):12076–90. doi: 10.1002/ece3.6830 (PMC7663989; doi:10.1002/ece3.6830)
Supplement: Supplementary file 2 — Appendix S1 [file ECE3-10-12076-s002.docx]

Appendix A. Additional habitat variables considered in Potential model development. These are members of the Coastal Geomorphology or Substratum classes. Shoreline conditions were projected out into adjacent open water Aquatic Habitat Areas (AHA).

| **Variable class** | **Variable** |
| --- | --- |
| **Geomorphology** | **Percentage as Bedrock (non-resistant); sedimentary** |
|  | **Percentage as Sandy beach/dunes; low to moderate erodibility** |
|  | **Percentage as High (>15m) bluff; cohesive, moderately** |
|  | **Percentage covered Sandy/silty banks; highly erodible.** |
|  | **Percentage as Clay banks; very cohesive, highly erodible.** |
|  | **Percentage as Artificial; areas where anthropogenic** |
|  | **Percentage as Bedrock (resistant)** |
|  | **Percentage as Semi-protected wetlands; protected by natural features such as baymouth barriers.** |
|  | **Percentage as Open Shoreline Wetlands; mostly emergent vegetation.** |
|  | **Percentage as Low Riverine/Coastal Plain; flood prone, low to moderate erodibility.** |
|  | **Percentage as Low (<15m) bluff; moderate erodibility.** |
|  | **Sinuosity of coastline (ratio of straight-line distance to shoreline distance)** |
|  | **Slope of the bottom** |
| **Substratum** | **Shoreline substratum Percentage as Sand** |
|  | **Shoreline substratum Percentage as Bedrock (non-resistant)** |
|  | **Shoreline substratum Percentage as Sand/gravel lag over clay** |
|  | **Shoreline substratum Percentage as Clay** |
|  | **Submerged substratum (Percentage of AHA as hard or soft)** |


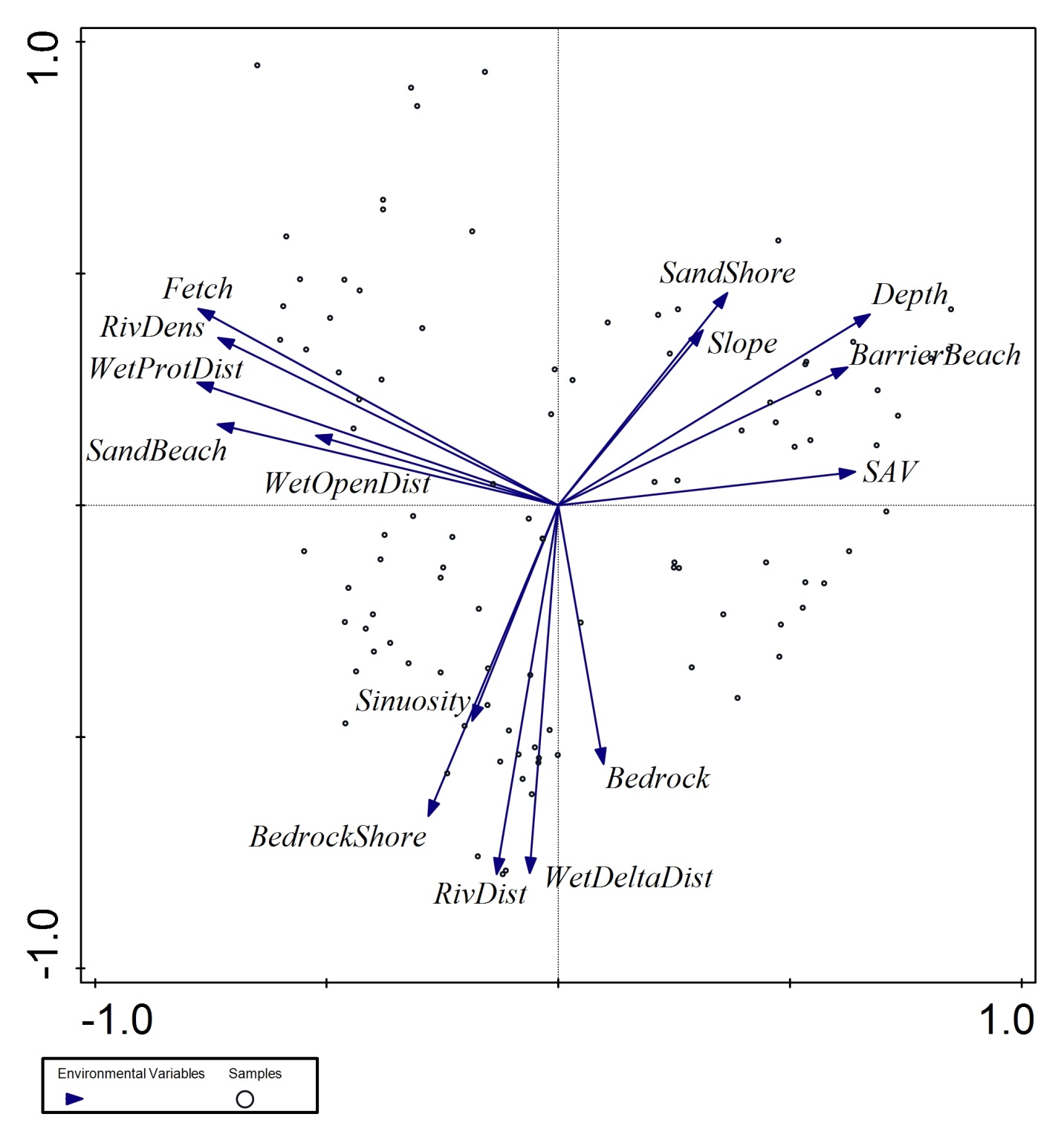
Figure. A1. The first two axes of the Principal Components Analysis. Samples are represented by circles. Vectors represent the direction of positive change in habitat values and vector length indicates the rate of change in value. See Table 1 and Appendix A table for variable definitions.
